# Redox Cycling in Two-Polarized Electrode Sensors: Diagnostic Insights Guided by Theory

**DOI:** 10.1021/acsmeasuresciau.6c00024

**Published:** 2026-03-20

**Authors:** Javier López-Asanza, Antonio Jesús Martínez-García, José Víctor Hernández-Tovar, Joaquín González, Angela Molina, Eduardo Laborda

**Affiliations:** Departamento de Química Física, Facultad de Química, Regional Campus of International Excellence “Campus Mare Nostrum”, 16751 Universidad de Murcia, 30100 Murcia, Spain

**Keywords:** signal enhancement, simulation tools, interelectrode feedback, cyclic voltammetry, chronoamperometry

## Abstract

Two-polarized electrode (2PE) electrochemical configurations are adopted in miniaturized and disposable sensors, reducing instrumentation complexity and avoiding practical complications associated with reference electrodes. Redox-cycling strategies have been proposed for signal amplification by preconverting the target species to complete a reversible redox couple, thereby enabling sustained interconversion between the two polarized electrodes. In these configurations, rational device design and quantitative interpretation of the measured response require explicit consideration of the mutual coupling between the two interfacial processes. This work develops a theoretical framework for redox cycling in two-electrode, single-potentiostat configurations, describing the full current–potential-time response under chronoamperometric and cyclic voltammetric conditions. Closed-form expressions are obtained for the current, interfacial concentrations and local potentials at each electrode. Working curves map the signal enhancement and defining features of chronoamperometric and voltammetric responses across the steady-state, transient redox-cycling and semi-infinite diffusion regimes, thereby providing practical diagnostic criteria for the design, characterization and interpretation of 2PE devices. The theory and signal-analysis protocols are validated experimentally, obtaining good agreement between simulated and measured responses.

## Graphical abstract

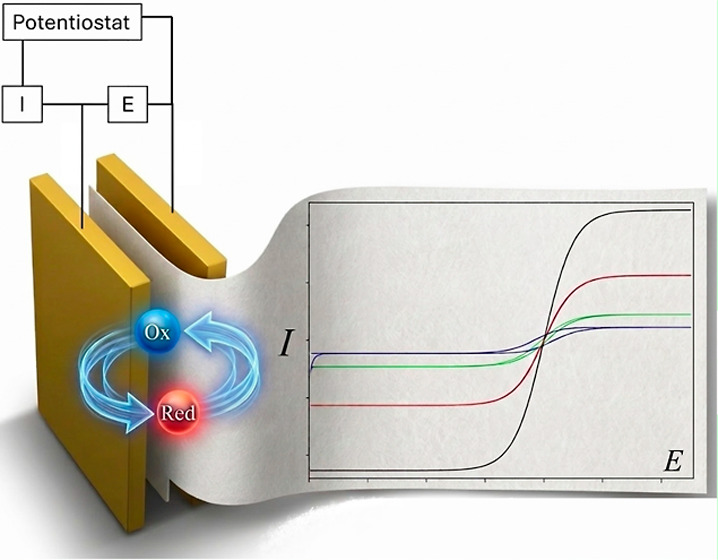

## 1 Introduction

The conventional three-electrode configuration (comprising a working, counter, and reference electrode) has long been the standard in electrochemical analysis due to its high precision in controlling and measuring electrode potentials. However, this setup presents inherent limitations for applications requiring device miniaturization, integration, and portability. In particular, the inclusion of a reference electrode often entails complex fabrication, susceptibility to drift or contamination,^1–3^ and issues related to liquid junctions,^3–5^ which complicate the design of compact or disposable electrochemical sensors. In contrast, systems employing two polarized electrodes (2PE) offer a compelling alternative. These configurations omit the reference electrode and instead operate by directly imposing a potential difference between two active electrodes.^6^ Such an approach not only reduces system complexity and cost but also facilitates the integration of electrochemical sensing into microfluidic platforms, point-of-care devices, and wireless or wearable technologies.^6–14^

Within the two-electrode framework, redox cycling has been proposed^13–19^ as a strategy to recover analytical sensitivity by exploiting the close proximity of two electrodes to repeatedly interconvert the oxidized, O, and reduced, R, forms of a reversible redox couple mediator; in other words, a feedback regime is established with overlapping of the hypothetical diffusion layers of each electrode (O and R in Figure 1), thereby amplifying the faradaic response in a variety of platforms. In contrast to bipotentiostatic generator–collector implementations, where a single mediator form is sufficient because the missing counterpart can be generated electrochemically,^20–27^ two-electrode and single-potentiostat operation generally requires that both O and R be available at startup to sustain complementary charge–transfer reactions at the two coupled interfaces. For analytical purposes, this constraint can be turned into an asset by introducing one mediator form and generating its counterpart at the expense of the analyte prior to readout, for example

$${Analyt\;e}_{red}+O\left(excess\right)\to {Analyt\;e}_{ox}+R$$

**Figure 1:**
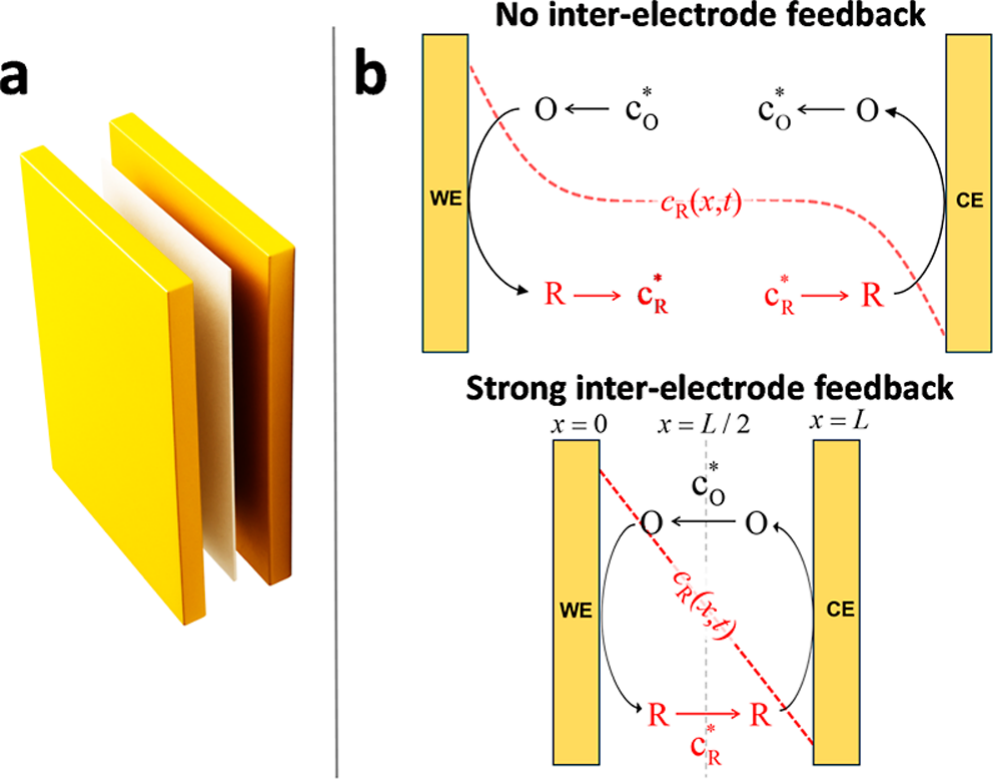
(a) Schematic illustration of the experimental assembly consisting of two parallel gold plates (acting as working and counter electrodes) separated by a filter paper layer. (b) Schematic representation of the modeled system consisting of two facing electrodes (WE and CE) separated by a distance *L*. A schematic concentration profile of species R (red dashed line) is included for clarity.

Among other examples reported, analyte–redox couple mediator systems include creatinine-ferri/ferrocynide^2^ and oxygen-4-quinoneimine/4-aminophenol.^13^ Hence, the electroanalytical response is decoupled from the target’s direct heterogeneous electron-transfer kinetics and from possible mechanistic complications (*e.g*., chemical irreversibility and/or coupled reactions) that may otherwise suppress cycling efficiency and analytical signal. Also, another common practical situation is the deliberate preloading of the mediator couple (i.e., both O and R introduced at defined ratios) to enable calibration and systematic performance assessment of sensing platforms.^2,13^

In spite of the advantages in terms of simplicity, cost, and integration, the theoretical treatment of two-electrode systems is more complex and, in general, scarcely explored.^2^ Two-electrode cells exhibit unique characteristics compared to conventional three-electrode setups due to the division of the driving force between the two polarized interfaces, leading to current responses that are fundamentally distinct from those observed in classical configurations. The electrochemical behavior is determined by the balance between the two coupled electrode processes, which modulates the shape, intensity and position of the signal.^6,28–30^ Consequently, specific theoretical formalisms and diagnostic criteria are required for their proper analysis and interpretation. In this sense, despite the relevance of redox cycling in two-electrode configurations without reference electrode for miniaturized and integrated sensors, analytical theoretical models have so far been limited to chronoamperometric measurements under limiting current^31^ and steady state^19,32,33^ conditions; other operating conditions and techniques have mainly been addressed by means of numerical simulations,^19,22,34–36^ which yield results of limited generality and do not allow for a truly a priori analysis and prediction of the sensor behavior.

In this study, an analytical theoretical framework is developed for redox cycling in two-electrode configurations to describe the current–potential–time response of reversible one-electron transfer processes involving solution-phase electroactive species. The theory is based on the derivation of analytical solutions for the concentration profiles and interfacial potentials under arbitrary potential-controlled perturbations, with particular emphasis on widely used techniques such as chronoamperometry, cyclic staircase voltammetry (CSV), and cyclic voltammetry (CV). The formulation encompasses both chronoamperometry at arbitrary applied potentials and cyclic voltammetry. Through a rigorous examination of the underlying boundary value problem, the analysis shows that the interfacial concentrations of the redox species remain time-independent under these conditions in which the two electrode processes are intrinsically coupled by charge balance, a common redox couple and a “shared” diffusion domain. This finding extends two major implications beyond the semi-infinite diffusion regime:^37,38^ First, the contributions of time (and geometry) and of the applied potential can be factorized, substantially simplifying the theoretical treatment; second, the superposition principle remains valid, allowing a sequence of potential steps to be rigorously represented as the sum of independent solutions for each individualstep. Mathematical expressions are also derived for the interfacial potential difference at each electrode, enabling separate analysis of electrode polarizations and thus identification of the rate-limiting electrode process in the electrochemical cell.^39^

Using the derived mathematical expressions, the influence of key system parameters (including electrode spacing, scan rate, diffusion coefficient and concentration ratio) is examined to rationalize their impact on the electrochemical signal. On this basis, robust guidelines are established for accurate analysis of experimental data. In addition, a web-based application implementing the theory is provided to enable simulations for a priori analysis and for fitting data. Experimental validation of the model is also provided, using a simple two-electrode redox cycling setup composed of two gold-coated glass plates and a thin nitrocellulose paper soaked in electrolyte solution. The close agreement between theoretical predictions and experimental chronoamperometry and voltammetry confirms the validity and applicability of the proposed theoretical framework.

## 2 Materials and Methods

Potassium ferricyanide (K_3_[Fe­(CN)_6_]), potassium ferrocyanide (K_4_[Fe­(CN)_6_]) and potassium chloride (KCl) were purchased from Sigma-Aldrich and used without further purification. All aqueous solutions were prepared using deionized water from a Milli-Q purification system (resistivity ≥18.2 MΩ·cm at 25 °C), with 0.1 M KCl as the supporting electrolyte.

Electrochemical measurements were conducted at room temperature using a BioLogic SP200 potentiostat (BioLogic Science Instruments). A two-electrode configuration was employed, consisting of two identical gold-coated glass plates, placed in a parallel, face-to-face arrangement similar to that proposed in ref,^27^ but eliminating the reference electrode entirely and relying solely on the imposed potential difference between the two polarized electrodes (see Figure 1a). These plates served as working (WE) and counter (CE) electrodes, enabling redox cycling. The interelectrode space was defined by a strip of filter paper (cellulose nitrate membrane, ca. 41 mm^2^, pore size 0.45 μm, Chmlab Group) saturated with the electrolyte solution. This paper fulfilled multiple roles: it acted as an ionic conductor, maintained a constant separation between electrodes, and crucially, prevented direct physical contact between them. The entire assembly was gently compressed to ensure uniform interfacial contact and reproducibility. Prior to each experiment, the system was equilibrated for several minutes to ensure uniform wetting of the paper matrix. To further improve measurement stability and minimize solvent evaporation, the outer edges of the paper and electrode assembly were sealed with silicone grease (LBSil 25, Labkem).

## 3 Theory

Let us consider the 2PE configuration depicted in Figure 1, where a single reversible redox couple, R/O, undergoes one-electron oxidation and reduction reactions at two polarizable electrode–solution interfaces of the same area, *A*. Both electrodes face each other at a fixed distance, *L*; when *L* is sufficiently small, the availability of both species at each electrode is affected by the other. The potential difference between the electrodes, *E*, is externally controlled. Under these conditions, the evolution of the redox concentrations with time (*t*) and position (*x*) relative to the electrodes is described by the following bvp

1 $$\begin{array}{l} \frac{\partial c_{R}\left(x,t\right)}{\partial t}=D\frac{\partial^{2}c_{R}\left(x,t\right)}{\partial x^{2}}\\ \frac{\partial c_{O}\left(x,t\right)}{\partial t}=D\frac{\partial^{2}c_{O}\left(x,t\right)}{\partial x^{2}} \end{array}$$

2 $$t=0,0\leq x\leq L:,c_{R}=c_{R}^{*},c_{O}=c_{O}^{*}$$

t>0,x=0:

3 $$\begin{array}{l} {\left(\frac{\partial c_{O}}{\partial x}\right)}_{x=0}=-{\left(\frac{\partial c_{R}}{\partial x}\right)}_{x=0}\\ c_{O}^{x=0}=e^{\eta_{WE}}c_{R}^{x=0} \end{array}$$

t>0,x=L:

4 $$\begin{array}{l} {\left(\frac{\partial c_{O}}{\partial x}\right)}_{x=L}=-{\left(\frac{\partial c_{R}}{\partial x}\right)}_{x=L}\\ c_{O}^{x=L}=e^{\eta_{CE}}c_{R}^{x=L} \end{array}$$

*c*_*i*_^*x* = 0^ and c_*i*_^*x* = *L*^ being the interfacial concentrations of species *i* at WE and CE, respectively, and

$$\begin{array}{l} \eta_{WE}=\frac{nF}{RT}\left(E_{WE}-E_{O/R}^{0́}\right)\\ \eta_{CE}=\frac{nF}{RT}\left(E_{CE}-E_{O/R}^{0́}\right) \end{array}$$ 5

where *n* is the number of electrons transferred in the electrode reaction (assumed to be concerted^40^).

The interfacial potentials at both electrodes are linked by the externally applied potential difference, *E*

6 $$E=E_{WE}-E_{CE}$$

and the current across the two interfaces must be the same

7 $$I=I_{WE}=I_{CE}$$

As demonstrated in the Supporting Information, attending to the linearity of the bvp, and via mathematical induction, it follows that the superposition principle holds in the context of redox cycling. Thus, for the application of a staircase series of potential steps (see Figure 2), *E*_1_, *E*_2_, ... *E*_p_, each applied for an equal time interval, τ, the current, *I*_p_, corresponding to any step *p* (ranging from 1 to *p*_max_) is given as the sum of products of a geometric-time factor, *z*_*m*_(*Λ*_CV_), and a potential dependent factor, *f*(η_m_)-*f*(η_m1_):

8 $${Ι}_{p}=nFAc_{R}^{*}\sqrt{\frac{Dv}{\pi \Delta E}}\sum_{m=1}^{p}\frac{z_{m}\left(\Lambda_{CV}\right)}{\sqrt{p-m+1}}\left(f\left(\eta_{m}\right)-f\left(\eta_{m-1}\right)\right)$$

where *v* = Δ*E*/τ is the scan rate (with Δ*E* being the step potential in absolute value)

$$\begin{array}{l} f\left(\eta_{0}\right)=0\\ f\left(\eta_{m>0}\right)=\frac{\varepsilon +1-\sqrt{{\left(\varepsilon +1\right)}^{2}-4\;\varepsilon \;\tanh^{2}\left(\eta_{m}/2\right)}}{2\tanh\left(\eta_{m}/2\right)} \end{array}$$ 9

with

10 $$\varepsilon =\frac{{c_{O}}^{*}}{{c_{R}}^{*}}$$

11 $$\eta_{m}=\frac{nF}{RT}E_{m},m=1,2...p$$

$$z_{m}\left(\Lambda_{CV}\right)=1+2\overset{\infty }{\underset{j=1}{\sum }}e^{-j^{2}\Lambda_{CV}/(4a\tau \left(p-m+1\right))},m=1,2...p$$ 12

13 $$\Lambda_{CV}=\frac{L^{2}a}{D}$$

14 $$a=\frac{Fv}{RT}$$

**Figure 2:**
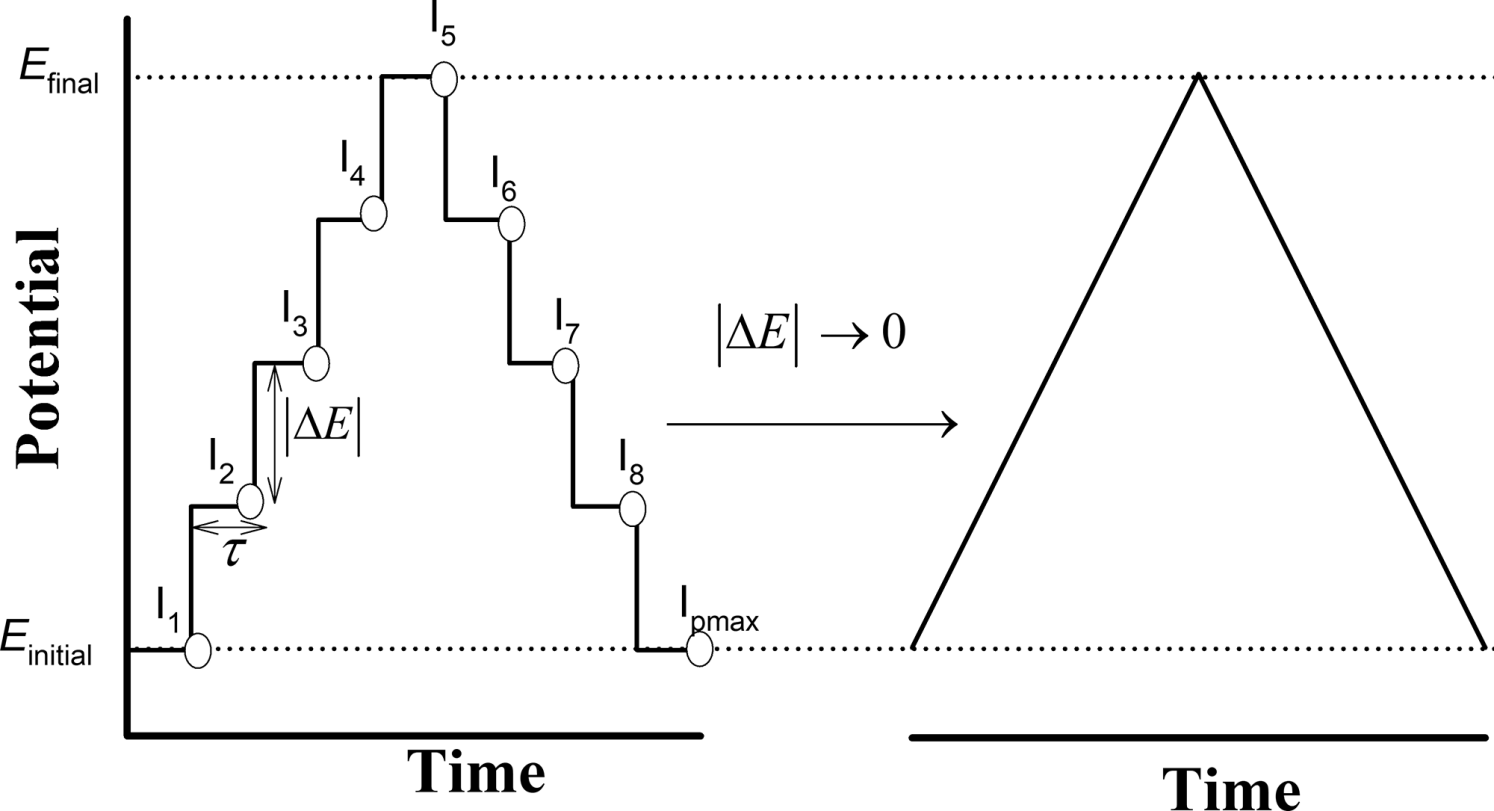
Schematic representation of the potential-time perturbations in CSV and CV. *Left*: CSV potential program defined by a potential increment Δ*E* and a step duration τ, indicating the discrete current measurements *I*_*p*_ taken at the end of each potential step. As Δ*E*→0, the staircase waveform approaches a continuous triangular potential sweep (*right*).

All other symbols have their usual meaning (see Table 1).

**Table 1:** List of Symbols Used in the Theoretical Formulation

symbol	definition
*A*	electrode area (m^2^)
*c*_O_*,*c*_R_*	initial concentrations of oxidized and reduced species (mol·m^–3^)
*c*_O_^*x* = 0^,*c*_R_^*x* = 0^	interfacial concentrations at WE (mol·m^–3^)
*c*_O_^*x* = *L*^,*c*_R_^*x* = *L*^	interfacial concentrations at CE (mol·m^–3^)
*D*	diffusion coefficient of redox species (m^2^·s^–1^)
*E*	applied potential: *E* = *E*_WE_-*E*_CE_ (V)
*E*_WE_,*E*_CE_	interfacial potential difference at WE and CE (V)
Δ*E*	potential step increment in CSV perturbation (V)
*F*	Faraday constant (C·mol^–1^)
*I*	current (A)
*I*_ss_	steady-state current (A)
*L*	interelectrode distance (m)
*n*	number of electrons transferred
*R*	gas constant (J·mol^–1^·K^–1^)
*t*	time (s)
*T*	absolute temperature (K)
*v*	scan rate in CSV and CV (V·s^–1^)
*x*	spatial coordinate (m)
δ	thickness of the linear diffusion layer (m)
ε	concentration ratio *c*_O_*/*c*_R_*
*Λ*_CA_	chronoamperometric dimensionless electrode-separation parameter
*Λ*_CV_	voltammetric dimensionless electrode-separation parameter
*Ψ*	dimensionless current in CSV and CV: $\Psi =I/\left(nFAc_{R}^{*}\sqrt{Da}\right)$

Note that the dimensionless parameter *Λ*_CV_ accounts for the influence of the interelectrode distance, being proportional to the square of the ratio between the finite and semi-infinite linear diffusion layers in cyclic voltammetry. Hence, a large value of *Λ*_CV_ indicates that the region over which each electrode perturbs the redox concentrations is small relative to the interelectrode separation; conversely, for small *Λ*_CV_ the interelectrode coupling becomes significant.

It is important to highlight that eq 8, derived for CSV, also provides the CV response when the potential increment is sufficiently small (Δ*E* < 0.01 mV).^41^ The corresponding implementation is freely accessible through the web-based applications available at https://redoxcycling-cvsim.streamlit.app/and https://redoxcycling-casim.streamlit.app/.

In addition to the voltammetric response, the methodology presented in the Supporting Information allows us to obtain analytical expressions for the interfacial concentrations and potentials, which offer valuable insights into the behavior of the system

15 $$\begin{array}{l} Working\;Electrode,\;\;\;\;\;\;\;\;\;Counter\;Electrode\\ c_{R}^{x=0\left(p\right)}=c_{R}^{*}\left(1-f\left(\eta_{p}\right)\right),c_{R}^{x=L\left(p\right)}=c_{R}^{*}\left(1+f\left(\eta_{p}\right)\right)\\ c_{O}^{x=0\left(p\right)}=c_{R}^{*}\left(f\left(\eta_{p}\right)+\varepsilon \right),c_{O}^{x=L\left(p\right)}=c_{R}^{*}\left(\varepsilon -f\left(\eta_{p}\right)\right) \end{array}$$

16 $$E_{WE}^{\left(p\right)}=E_{O/R}^{0́}+\frac{RT}{nF}\ln\left(\frac{f\left(\eta_{p}\right)+\varepsilon }{1-f\left(\eta_{p}\right)}\right),E_{CE}^{\left(p\right)}=E_{O/R}^{0́}+\frac{RT}{nF}\ln\left(\frac{\varepsilon -f\left(\eta_{p}\right)}{1+f\left(\eta_{p}\right)}\right)$$

It is important to highlight that the normalized current and the interfacial magnitudes depend on both ε and applied potential through the function *f*(η). Since ε is time-independent, the function *f*(η) is also constant over time. As a result, the normalized current, surface concentrations and interfacial potentials are determined solely by the concentration ratio of the two species and the applied potential, and they do not depend on the distance between the electrodes, *L*, similarly to that found when a three-electrodes cell is employed.^41^

### 3.1 Particular Cases

#### 3.1.1 Single Potential Step: Chronoamperometry

By making *p* = 1 in eq 8, the following expression for the current signal in chronoamperometry is derived

17 $$I=\frac{nFA{c_{R}}^{*}D}{\delta \left(\Lambda_{CA}\right)}f\left(\eta \right)$$

where δ­(*Λ*_CA_) denotes the thickness of the diffusion layer^42^

18 $$\delta \left(\Lambda_{CA}\right)=\frac{\sqrt{\pi Dt}}{1+2\sum_{j=1}^{\infty }e^{-j^{2}\Lambda_{CA}/4}}$$

with

19 $$\Lambda_{CA}=\frac{L^{2}}{Dt}$$

Note that the exponential function in eq 18 depends on parameter *Λ*_CA_ (instead of *Λ*_CV_), which also corresponds to the square of the ratio between the finite and semi-infinite diffusion layers when a constant potential is applied. Because diffusion layers grow with time, the dimensionless electrode-separation parameter is inherently time-scale dependent, and its numerical value is set by the characteristic time of the technique (i.e., the pulse duration in CA, eq 19, and the scan rate in CV, eq 13).

From eq 17, analytical expressions for the limiting currents at very positive and negative applied potentials can be derived. For *ε* > 1, the corresponding expressions are given by

20 $$\begin{array}{l} \lim_{\eta \to \infty }I=\frac{nFA{c_{R}}^{*}D}{\delta \left(\Lambda_{CA}\right)}\\ \lim_{\eta \to -\infty }I=-\frac{nFA{c_{R}}^{*}D}{\delta \left(\Lambda_{CA}\right)} \end{array}$$

whereas for *ε* < 1 the limiting current expressions tend to

21 $$\begin{array}{l} \lim_{\eta \to \infty }I=\frac{FA{c_{O}}^{*}D}{\delta \left(\Lambda_{CA}\right)}\\ \lim_{\eta \to -\infty }I=-\frac{FA{c_{O}}^{*}D}{\delta \left(\Lambda_{CA}\right)} \end{array}$$

These expressions show that the current is always governed by the less abundant species, and that the limiting electrode (WE or CE) depends on the applied potential.

#### 3.1.2 No Interelectrode Feedback

*Λ*_CV_ > 500 For large values of *Λ*_CV_, the term *z*_*m*_(*Λ*_CV_) approaches 1, $\delta \left(\Lambda_{CA}\right)\to \sqrt{\pi Dt}$ and the system reaches the semi-infinite linear diffusion response

22 $${Ι}_{p}=nFAc_{R}^{*}\sqrt{\frac{Dv}{\pi \Delta E}}\sum_{m=1}^{p}\frac{f\left(\eta_{m}\right)-f\left(\eta_{m-1}\right)}{\sqrt{p-m+1}}$$

Inspection of eq 22 reveals that the CV response in a two-electrode configuration scales with the square root of the scan rate (*v* ^1^′^2^), analogously to conventional three-electrode systems, given that all terms in the summation depend only on the applied potential factor, *f*(η_*m*_)-*f*(η_*m*‑1_), since *z*_*m*_(Λ_CV_)→1.

#### 3.1.3 Strong Interelectrode Feedback

*Λ*_CV_ < 3 For small values of *Λ*_CV_ (<3 for the current at *E* = 0 being negligible, specifically, less than 5% of the plateau current), the current signal becomes time-independent and the corresponding steady state current–potential response is sigmoidal. Under these conditions, the steady-state current can be described by the following expression

23 $$I_{ss}=\frac{nFADc_{R}^{*}}{\delta_{ss}}f\left(\eta \right)$$

with

24 $$\delta_{ss}=\frac{L}{2}$$

Equation 23 shows that the steady-state signal is governed solely by the applied potential and varies inversely with *L*. Hence, reducing the interelectrode gap increases the signal enhancement arising from redox cycling, because the reactant for each electrode reaction is regenerated over a shorter distance at the opposing electrode.

## 4 Results and Discussion

### 4.1 Chronoamperometry

According to eq 17, the potential and time dependences of the system’s current response can be separated. The potential-dependent component is governed by the ratio between the concentrations of electroactive species, *ε*, which determines the relative capacity of both electrodes to sustain the electric current; meanwhile, the time-dependent factor is determined by the interelectrode distance, parametrized by *Λ*_CA_. With respect to the latter, Figure 3a shows the influence of *Λ*_CA_ on the dimensionless current normalized relative to the case where semi-infinite diffusion holds (i.e., no redox cycling). Thereby, Figure 3a provides a working curve for anticipating the signal enhancement due to the redox cycling or, alternatively, for extracting the interelectrode spacing from straightforward chronoamperometric experiments. **Zone I**. No Interelectrode Feedback. For large *Λ*_CA_ (>21 for differences smaller than 1%), the experimental response becomes independent of *L* and follows a Cottrell-like decay with $1/\sqrt{t}$

**Figure 3:**
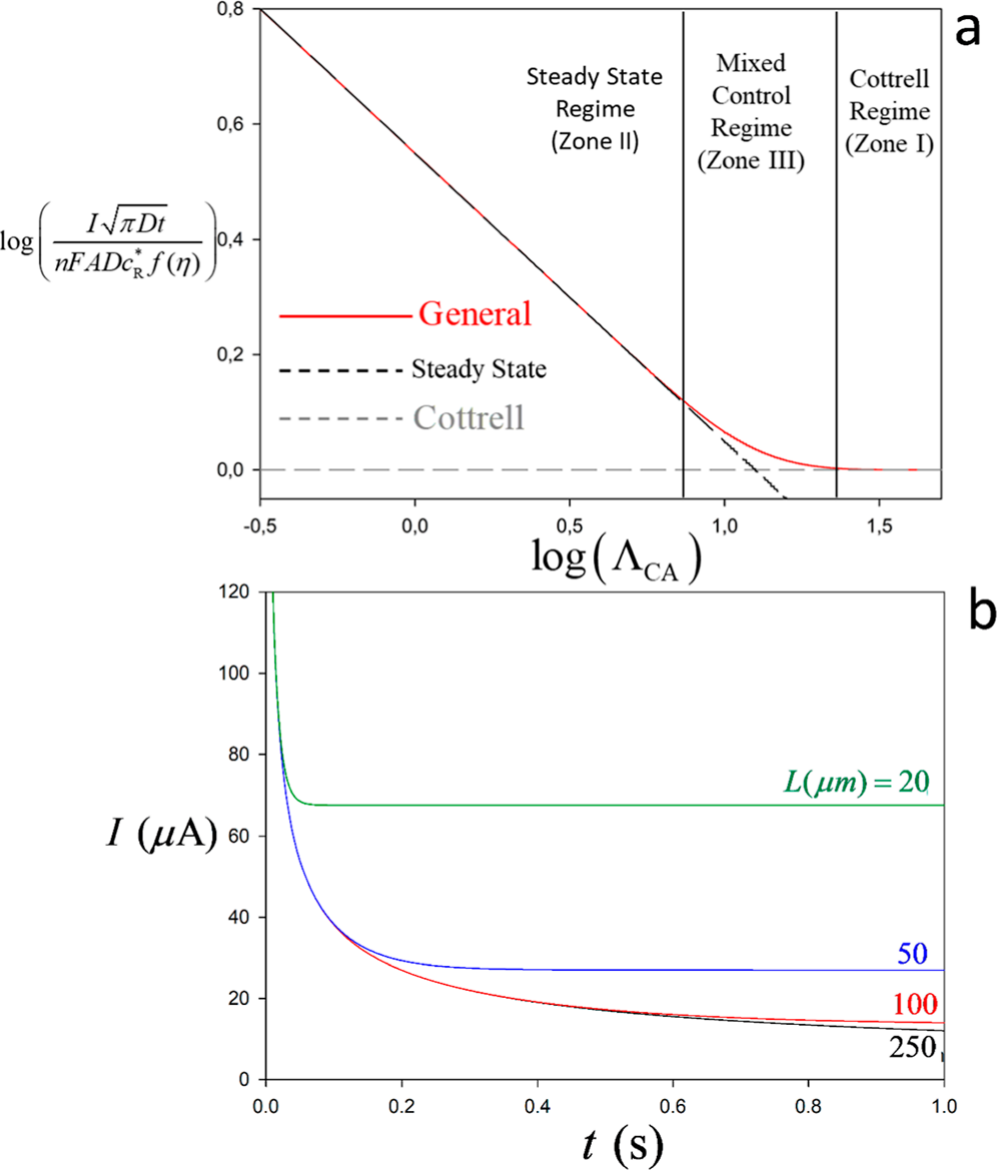
(a) Variation with *Λ*_CA_(eq 17) of the signal enhancement by redox cycling defined as the current signal relative to that in the absence of redox cycling (eq 25); (b) theoretical limiting-current chronoamperometric response (eq 20 or (21)) for different interelectrode distances, *L*. Parameters: *n* = 1, *c*_O_ = 1 mM, *c*_R_ = 1 mM (*ε* = 1), *D* = 1 × 10^–9^ m^2^ s^–1^, *A* = 7 × 10^–6^ m^2^, and *E* = 0.4 V.

25 $$I_{large\;L}=nFA{c_{R}}^{*}\sqrt{\frac{D}{\pi t}}f\left(\eta \right)$$

**Zone II**. Strong Interelectrode Feedback. For small *Λ*_CA_-values (<8 for differences below 1%), the system reaches the steady-state regime where the current is inversely proportional to *L*, allowing for strong signal amplification. Namely, from eqs 23 and 25 it can be readily concluded that such enhancement will be beyond 1 order of magnitude for $L<\sqrt{\pi Dt}/5$ , which means interelectrode distance below ca. 10 μm under typical experimental conditions

$$\left(D∼10^{-9}m^{2}/s,t∼1s\right)$$

**Zone III**. Intermediate interelectrode feedback (mixed regime, 8<Λ_CA_ < 21): An intermediate situation emerges in which the response is neither fully steady-state nor entirely governed by semi-infinite linear diffusion. Instead, the current is time-dependent yet exceeds the value predicted by eq 25 owing to the redox cycling.

The transition between the above three regimes is illustrated in dimensional form in Figure 3b. Note that analyzing chronoamperometry across different time domains provides additional information. Thus, at short times, Cottrell behavior dominates, allowing the concentration of the limiting species or its diffusion coefficient to be determined (eq 25). At longer times, once steady-state is reached, the current scales with 2/*L* (eq 23); if the remaining parameters are known, this relationship enables the determination of the interelectrode distance from the steady-state current. Alternatively, the full chronoamperometric response can be analyzed using the chronoamperometric simulation framework here provided, allowing one- or two-parameter fitting procedures to be applied depending on the experimental constraints.

### 4.2 Cyclic Voltammetry

In analogy with CA, eq 8 indicates that the CV response is governed by a time–geometric parameter, *Λ*_CV_, as well as by the concentration ratio between the oxidized and reduced forms, ε.

Figure 4a,b show the influence of *Λ*_CV_ on the semidimensionless CV curves when the concentrations of the oxidized and reduced forms are equal (*ε* = 1). When *Λ*_CV_ is small (Figure 4a), the system operates under strong redox cycling mode and attains steady-state. Under these conditions, the CV response is sigmoidal, the current is independent of the scan rate, and it scales as 2/*L* (eq 23); consequently, larger currents and greater analytical sensitivity can be achieved by reducing the spacing between electrodes.

**Figure 4:**
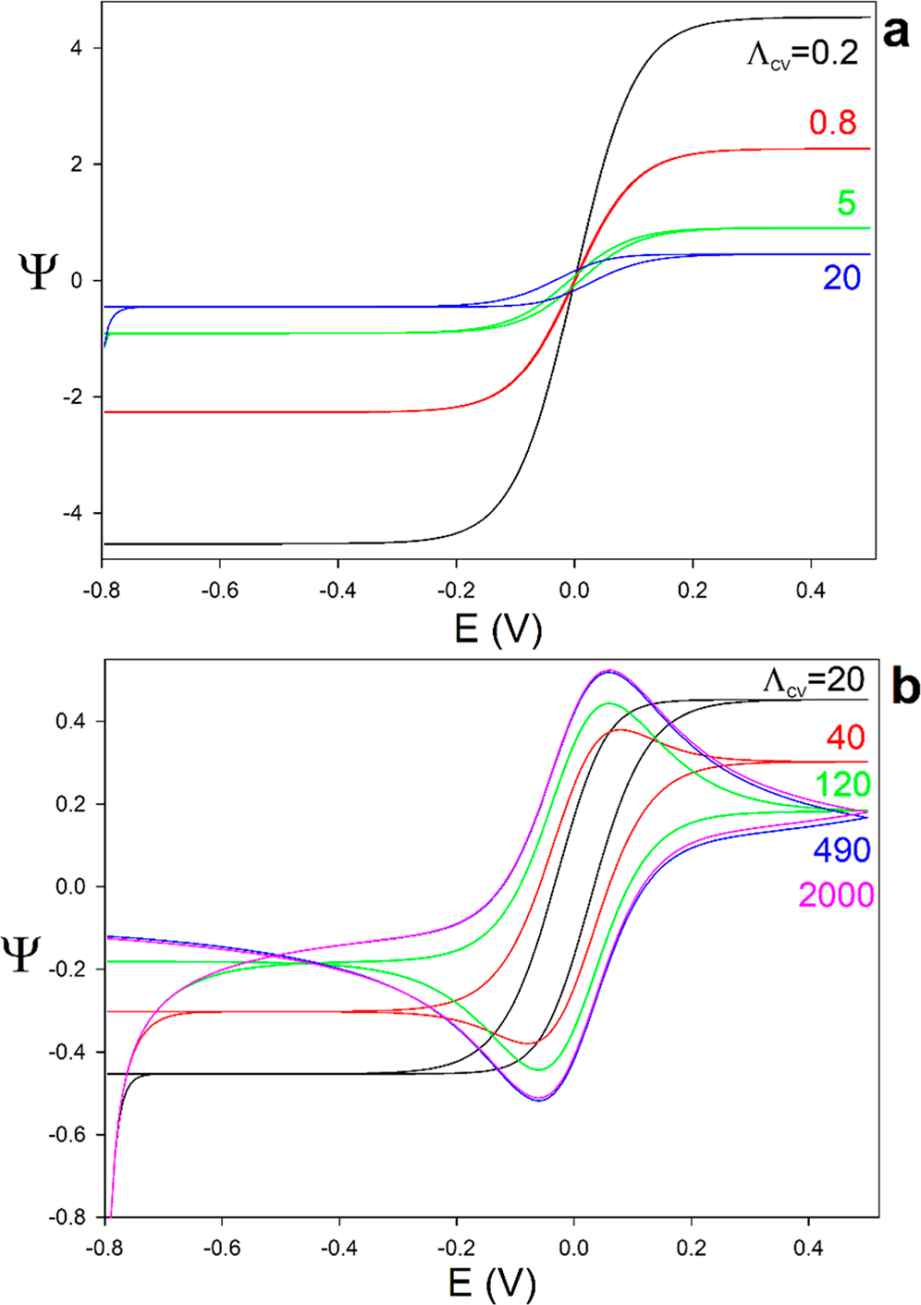
(a,b) Influence of *Λ*_CV_ on the CV response (eq 8) for *ε* = 1. (a) *Λ*_CV_ ranging from 0.2 to 20, illustrating the transition from strong to intermediate feedback; (b) *Λ*_CV_ from 20 to 2000, covering the transition from intermediate to negligible interelectrode feedback. Δ*E* = 10^–4^V.

As *Λ*_CV_ increases with increasing the interelectrode spacing and/or the scan rate, the system enters the intermediate redox-cycling regime (see green and blue lines in Figure 4a). Thus, the CV response exhibits a sigmoidal shape, approaching a quasi-steady-state, but the signal is not entirely time-independent so that the current–potential curves of the forward and reverse scans do not overlap. Upon further increasing *Λ*_CV_, Figure 4b, well-defined positive and negative peaks emerge and the response gradually transitions toward the no-feedback regime (see pink and blue lines in Figure 4b). Under these conditions, there exists a current ‘tail’ at the beginning of the experiment (most negative potentials) associated with the reduction of O at the working electrode and the simultaneous oxidation of R at the counter electrode. Note that this tail is similar to that encountered in conventional three-electrode measurements when the complete redox couple is present.^41^ This arises from the fact that both redox forms (O and R) are initially present in solution. Thus, as soon as the imposed interelectrode potential difference deviates from the equilibrium condition (*E* = 0), a finite driving force is established that biases reduction at one electrode and oxidation at the other. Under transient conditions (Figure 4b), this manifests as the above-mentioned ‘tail’, reflecting the time-dependent concentration gradients.

The effect of parameter ε is investigated in Figure 5, where its influence is illustrated under the three distinct regimes. It is assumed that species R remains at the same or a lower concentration than species O. Consequently, species R will be considered the limiting factor for the current response, and ε will always be treated as *ε* ≥ 1.

**Figure 5:**
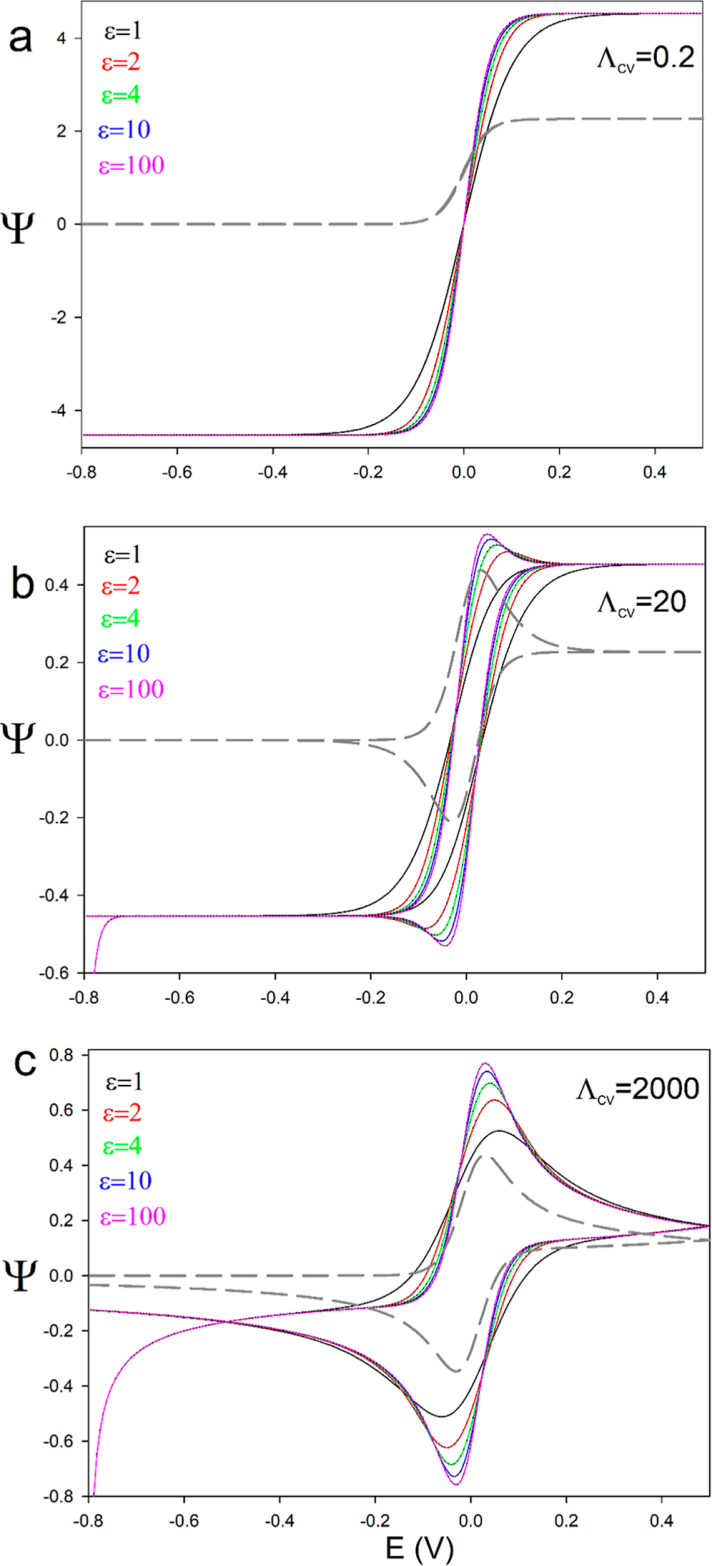
Influence of *ε* on the CV response (eq 8) under the different regimes: (a) *Λ*_CV_ = 0.2 (strong feedback), (b) *Λ*_CV_ = 20 (intermediate feedback), and (c) *Λ*_CV_ = 2000 (no feedback). For comparison, the theoretical response predicted for a bipotentiostat single-species configuration have been included (gray dashed line).^43^

Under strong redox cycling (Figure 5a), the parameter *ε* does not affect either the plateau magnitudes or the position of the CV response, the half-wave potential remaining at 0 V, as can be rigorously deduced from eq S31. On the other hand, the *ε*-value has an influence on the shape of the voltammetric wave: As *ε* increases, the response becomes progressively steeper, and the limiting currents are reached at potentials closer to 0 V. Hence, *ε* can be estimated from the difference between the three-quarter- and one-quarter-wave potentials, *E*_3/4_ - *E*_1/4_ (eq S35). The variation with ε spans from $\frac{RT}{F}\ln\left(81\right)$ at *ε* = 1 (approximately 113 mV at 298 K) to $\frac{RT}{F}\ln\left(9\right)$ at sufficiently large *ε* values (approximately 57 mV).

For comparison, the steady-state response predicted for strong redox cycling in a bipotentiostat configuration starting from a single redox form, analogous to scanning electrochemical microscopy, SECM, under positive feedback, is also shown (gray dashed lines). Provided the same interelectrode distance *L*, the steady-state current in the SECM mode scales as 1/*L* and is therefore half that in the 2PE configuration, which scales as 2/*L*. With regard to the steepness of the voltammetric wave, the *E*_3/4_ - *E*_1/4_-value in SECM is $\frac{RT}{nF}\ln\left(9\right)$ , which coincides with the limiting value approached in the 2PE configuration at large *ε*. As discussed in Figure S1, this behavior is related to the fact that, for sufficiently large *ε*, the electrode associated with the nonlimiting process effectively behaves as nonpolarizable.

Under intermediate feedback conditions (Figure 5b), the value of *ε* affects the shape of the voltammograms, which varies from sigmoidal for *ε* close to 1 to peak-shaped when *ε* is increased; eventually, the voltammograms (almost) overlap for *ε* > 10. Therefore, highly unequal initial concentrations (*ε* > 10) ‘hinder’, but not suppress, the attainment of the sigmoidal voltammograms characteristic of the strong feedback regime (see Figure 4). Thus, while the peak vanishes for *Λ*_CV_(=*L* ^2^ *a*/*D*) < 30 when *ε*∼1 (Figure 4b), it does for *Λ*_CV_ < 15 when *ε* > 10. In practice, for conventional values of diffusion coefficient (∼10^–9^ m^2^/s), this corresponds to scan rates in the range of 4–8 V/s for *L*∼10 μm and of 40–80 mV/s for *L*∼100 μm. Again, this influence of *ε* can be rationalized in terms of the interfacial polarizations at the two electrodes: When one redox form is present in large excess (*ε* > 10), the ‘local’ scan rate at the limiting interface varies more rapidly, ca. twice faster, in the situation where there is large excess of one redox form (see Figure S1). Consequently, for *ε* > 10 the system reaches the sigmoidal response at longer time-scale and/or shorter interelectrode distance.

The change in the voltammogram shape in this intermediate regime can be exploited to estimate the values of both the diffusion coefficient and the interelectrode distance. Thus, for a given experimental system, identifying the scan rate at which the voltammogram becomes sigmoidal, enables us to determine the ratio $\frac{L^{2}}{D}$ (=15/*a* for *ε* > 10 or 30/*a* for *ε*∼1). Then, the plateau currents of the sigmoid obtained at sufficiently slow scan rates yield the ratio $\frac{D}{L}=\frac{I_{plateau}}{2nFAc_{R}^{*}}$ Finally, combining these two ratios yields separate estimates of *D* and *L*. Alternatively, the full voltammetric response can be analyzed using the simulation framework here established.

Within the no-feedback regime (Figure 5c), and consistent with the behavior of the interfacial potentials discussed above (Figure S1), increasing the concentration ratio ε amplifies the voltammetric signal (by 15–30%, depending on the initial and vertex potentials) and a markedly reduces the peak-to-peak separation, which approaches the ca. 60 mV value expected for three-electrode setups and the SECM mode (gray dashed line).

It is worth noting that, under all conditions, the CV remains centered at 0 V and the forward and reverse peaks (when present) appear symmetrically about 0 V. These characteristics provide useful diagnostic criteria for verifying appropriate 2PE operating conditions.

The effects of Λ_CV_ and ε discussed above can be generalized and visualized more systematically through the working curves for the maximum dimensionless current, *Ψ*_max_, and the peak-to-peak separation, Δ*E*_pp_, given in Figure 6. These working curves provide practical guidance for identifying the operational regime, optimizing experimental conditions to maximize signal quality, or assessing the reversibility of a redox couple.

**Figure 6:**
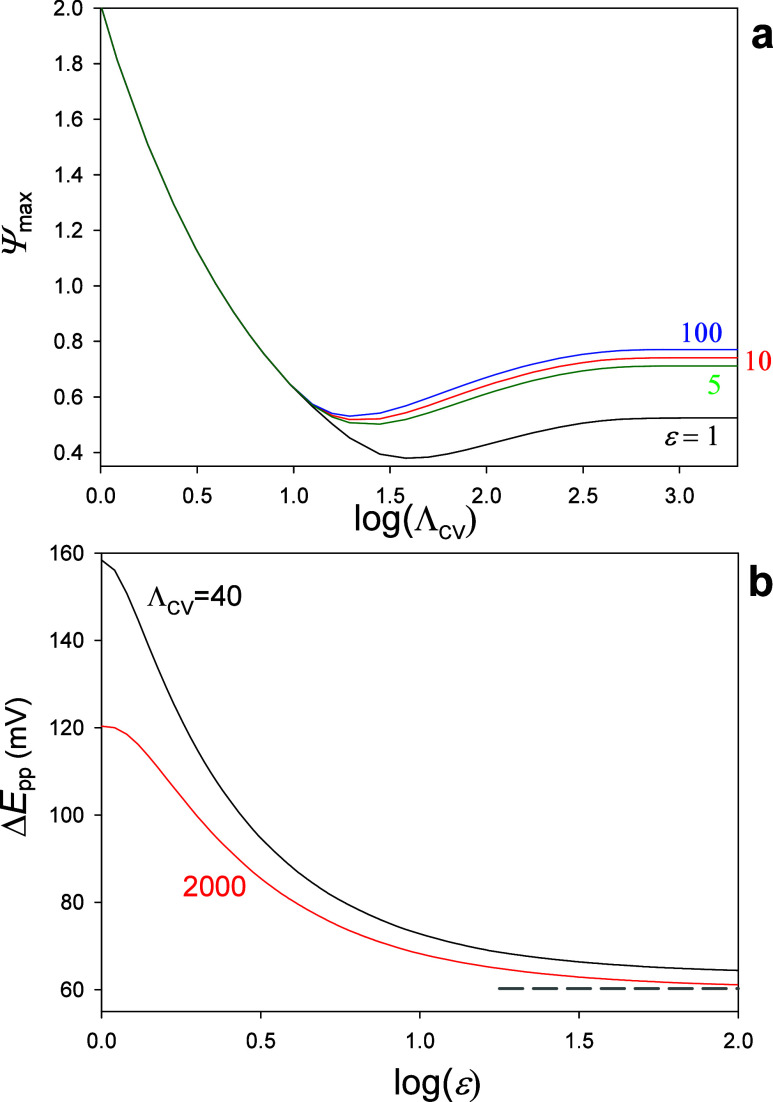
(a) Variation of the dimensionless maximum current, $\Psi_{\max}\left(=I_{\max}/\left(FAc_{R}^{*}\sqrt{Da}\right)\right)$ , as a function of log­(*Λ*_CV_) for different *ε* values. (b) Variation of the peak-to-peak separation, Δ*E*_pp_, with log­(ε) for two representative *Λ*_CV_ values corresponding to the mixed (black line) and the semi-infinite diffusion (red line) regimes. For reference, the theoretical Δ*E*_pp_-value for a bipotentiostat single-species configuration is also shown (gray dashed trace).

With regard to the variation of *Ψ*_max_ (Figure 6a), for log­(*Λ*_CV_) smaller than 1.1, *Ψ*_max_ increases as *Λ*_CV_ is decreased (eq 12) and the curves corresponding to different *ε*-values coincide since the system operates under steady-state where the *plateau* current is independent of ε (see Figure 4a). For slightly larger log­(*Λ*_CV_) values, the curves start to diverge for different *ε* values, which marks the onset of the intermediate regime. As the system approaches the no-feedback case, the signal increases with log­(*Λ*_CV_) and subsequently stabilizes at the semi-infinite diffusion limiting value. In this regime, the experimental current becomes proportional to $\sqrt{v}$ , as in conventional three-electrode setups and in two-electrode configurations where the interelectrode feedback is negligible.^29^

Figure 6b shows how the peak-to-peak separation varies with the concentration ratio for two representative values of *Λ*_CV_ = 40 (intermediate feedback) and*Λ*_CV_ = 2000 (no-feedback). In both cases, the peak-to-peak separation changes markedly for moderate *ε* values, and it becomes nearly constant when there is a large excess of the oxidized species (*i.e*., large *ε*).^29^ Specifically, in the intermediate regime (black line), the Δ*E*_pp_-value is around 158 mV for *ε* = 1 and decreases markedly to about 64 mV at large *ε*. In the no-feedback regime (red line), a similar trend is observed, with the separation dropping from approximately 120 mV to around 60 mV. Note that experimental Δ*E*_pp_ larger that these reference values can serve to indicate limitations arising from sluggish electron transfer and/or ohmic losses.

### 4.3 Experimental Validation

To verify the theoretical predictions and data-analysis protocols, a series of experiments were performed using a solution containing potassium ferrocyanide (0.72 mM) and potassium ferricyanide (0.54 mM). As noted in the Introduction, in practice the corresponding concentration ratio can be set deliberately (for example, to calibrate the interelectrode configuration, as done here) or it can arise from a well-defined conversion of an analyte that generates the complementary mediator form prior to measurement.^2,12,13^ The 2PE configuration included two gold-coated glass plates connected directly to the potentiostat. Care was taken to prevent physical contact between the electrodes, using a strip of nitrocellulose paper as the matrix where the electrolyte solution is contained.

Figure 7a shows the experimental limiting current (solid lines) together with the best-fit theoretical response from the model (points). As predicted, the current initially follows Cottrell behavior and progressively decreases until reaching a constant steady-state value of approximately 23 μA. Following the procedure described in Section 4.1, the diffusion coefficient was estimated from the short-time Cottrellian region, yielding *D* = 7.6 × 10^–10^m^2^ s^–1^. Subsequently, using the steady-state current and the value of *D*, an interelectrode distance of *L* = 147 μm was obtained from $L=\frac{2nFDAc_{R}^{*}}{I_{ss,\lim}}$ . In addition, a two-parameter fit of the full chronoamperogram was performed, giving *D*=(7.3 ± 0.2)×10^–10^m^2^s^–1^and *L* = 156 ± 2 μm. These values are consistent with those obtained from the independent short- and long-time analyses, supporting the internal coherence of the approach.

**Figure 7:**
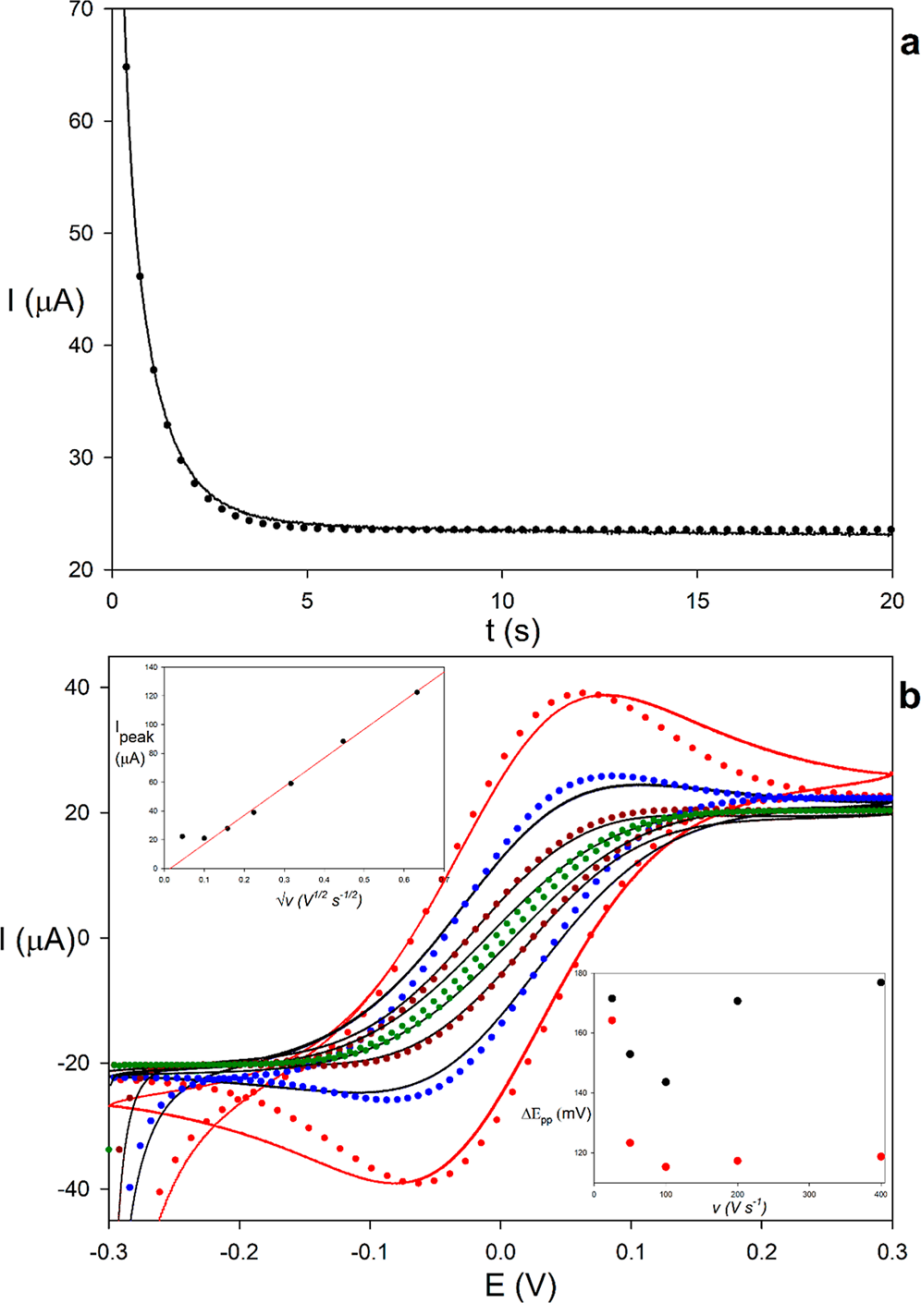
Experimental (solid lines) and theoretical (points) (a) chronoamperometry and (b) cyclic voltammograms recorded at scan rates of 2 (green), 10 (brown), 25 (blue) and 50 (red) mV·s^–1^ for the 2PE redox cycling system described in Section 2 using ferricyanide/ferrocyanide as redox pair (0.54 and 0.72 mM, respectively). The upper inset shows the experimental maximum current obtained at scan rate from 2 to 400 mV·s^–1^ vs $\sqrt{v}$ ; the lower inset shows the experimental (black points) and theoretical (red points) peak-to-peak separation *vs v*.

Figure 7b displays the experimental CV (solid lines) under different feedback regimes by varying the scan rate while keeping the interelectrode distance fixed. As the scan rate is decreased, the voltammogram transitions from a peaked response at the highest scan rates (e.g., red line) to the sigmoidal curve characteristic of (almost) steady-state conditions (green line). Regarding the effect on signal magnitude (see inset of Figure 7b), at the highest *v*-values the current increases proportionally to the square root of the scan rate, consistent with the absence of interelectrode feedback. As the scan rate is reduced and the system approaches the positive feedback regime, this proportionality is gradually lost. Across the entire range of scan rates investigated, a clear symmetry between the forward and reverse peaks is preserved, with the midpoint potential remaining at 0 V within ±1 mV in all cases.

As discussed in Section 4.2, closer inspection of the transition in shape can be used to extract information about, and to characterize, the system. Thus, the disappearance of well-defined peaks in the experimental voltammetry takes place at ca. 25 mV/s. As mentioned in CV section, this should correspond to *Λ*_CV_ around 30 as the experimental *ε* is close to unity, which yields an experimental value of *L* ^2^/*D* ≈ 30.8 s. From the experimental steady state *plateau* current (ca. 22 μA) a value of *L*/*D* ≈ 1.9 × 10^5^ s/m is obtained with eq 23. Combining these two results, estimations of the interelectrode distance and the diffusion coefficient in the paper matrix can be readily extracted: *L* ≈ 162 μm and *D* ≈ 8.5 × 10^–10^ m^2^/s. These estimates are in reasonable agreement with those obtained from the more rigorous least-squares analysis of the full experimental CVs recorded over 2–25 mV/s, *L* = 155 ± 3 μm and *D* = (7.2 ± 0.2)× 10^–10^ m^2^·s^–1^, as well as with the CA analysis and literature diffusion coefficient reported for comparable paper-based matrices.^200^

While a strong agreement is observed between the theoretical predictions and the experimental results for scan rates below 50 mV*s* ^–1^, increasing discrepancies emerge at higher scan rates. These deviations may be attributed to the quasi-reversibility of the ferro/ferri redox couple^44^ and/or uncompensated resistance, both of which become more pronounced as the scan rate increases. Thus, the model predicts that the peak-to-peak separation decreases with increasing scan rate and reaches a minimum value (lower inset in Figure 7b). This trend is in line with the experimental behavior at low scan rates; at higher scan rates, however, the experimental decrease is less pronounced than predicted, with peak-to-peak separations remaining systematically larger.

## 5 Conclusions

A comprehensive theoretical framework has been developed for interpreting the current–potential-time response of reversible redox cycling systems in two-polarized electrode (2PE) with single potentiostat control. Even under semi-infinite diffusion conditions, the conventional criteria for three electrode setups may not hold as a result of the coupled charge transfers at two polarized interfaces in series and the intrinsically different distribution/partition of interfacial potentials, which can be inspected with the theory here presented.

The analytical expressions reported for the current, interfacial concentrations and local potentials at each electrode enable to anticipate and rationalize the signal enhancement across the distinct regimes. For this, working curves and diagnosis criteria are provided. Specifically, the chronoamperometric and transient cyclic voltammetric responses offer valuable information and straightforward criteria for rapidly identifying the interelectrode feedback regime. Moreover, they enable simple, simultaneous estimation of the interelectrode spacing and the diffusivity of the redox mediator (from the scan-rate range over which the response transitions from sigmoidal to peaked behavior), while also revealing potential limitations arising from sluggish electron-transfer kinetics or resistive losses within the sensor architecture (from deviations from the predicted peak-to-peak separation). For more detailed exploration and more accurate parameter extraction, the theoretical solutions have been integrated into web-based simulation apps.

The theoretical predictions and data-analysis protocols have been validated using a simple 2PE platform consisting of two facing gold-coated plates separated by a thin, electrolyte-soaked nitrocellulose paper containing a ferri/ferrocyanide redox couple deliberately preloaded at a defined concentration ratio for benchmarking of the interelectrode configuration. This configuration yielded chronoamperometric and voltammetric responses in good agreement with the analytical model.

## Supplementary Material



## Data Availability

Note that this tail is similar to that encountered in conventional three-electrode measurements when the complete redox couple is present.

## Notes

Mathematical formulation and resolution of the boundary value problem of redox cycling in two-polarized electrode systems; Interfacial potentials and concentrations; Theoretical concentration profiles in chronoamperometry and cyclic voltammetry (PDF)

## References

[1] Biswas N. B., Read T., Levey K. J., Macpherson J. V. (2025). Choosing
the Correct Internal Reference Redox Species for Overcoming Reference
Electrode Drift in Voltammetric PH Measurements. ACS Electrochem..

[2] Bezinge L., Tappauf N., Richards D. A., Shih C.-J., deMello A. J. (2023). Rapid Electrochemical
Flow Analysis of Urinary Creatinine on Paper: Unleashing the Potential
of Two-Electrode Detection. ACS Sens..

[3] Troudt B. K., Rousseau C. R., Dong X. I. N., Anderson E. L., Bühlmann P. (2022). Recent Progress
in the Development of Improved Reference Electrodes for Electrochemistry. Anal. Sci..

[4] Handbook of Reference Electrodes; Inzelt, G. , Lewenstam, A. , Scholz, F. , Eds.; Springer Berlin Heidelberg: Berlin, Heidelberg, 2013; . 10.1007/978-3-642-36188-3.

[5] Kimmel D. W., LeBlanc G., Meschievitz M. E., Cliffel D. E. (2012). Electrochemical
Sensors and Biosensors. Anal. Chem..

[6] Xiong J., White H. S. (2013). The i–V Response
of an Electrochemical Cell
Comprising Two Polarizable Microelectrodes and the Influence of Impurities
on the Cell Response. J. Electroanal. Chem..

[7] Santhiago M., Kubota L. T. (2013). A New Approach for
Paper-Based Analytical Devices with
Electrochemical Detection Based on Graphite Pencil Electrodes. Sens. Actuators B Chem..

[8] Gutiérrez-Capitán M., Balada E., Aviñó A., Vilaplana L., Galve R., Lacoma A., Baldi A., Alcamí A., Noé V., Ciudad C. J., Eritja R., Marco M.-P., Fernández-Sánchez C. (2025). Unraveling the Amplification-Free
Quantitative Detection of Viral RNA in Nasopharyngeal Swab Samples
Using a Compact Electrochemical Rapid Test Device. Anal. Chem..

[9] Nagar B., Silva W. O., Girault H. H. (2021). Voltammetry in Two-Electrode
Mode
for Rapid Electrochemical Screening Using a Fully Printed and Flexible
Multiplexer Sensor. ChemElectroChem.

[10] Park S., Kim J., Park S., Kim Y., Yang H. (2025). Electrochemical Immunosensor
Employing a Potential-Guiding, Kinetically Robust, and Counterbalancing
Redox Mediator in a Two-Electrode Configuration. ACS Sens..

[11] Gutiérrez-Capitán M., Sanchís A., Carvalho E. O., Baldi A., Vilaplana L., Cardoso V. F., Calleja A., Wei M., de la Rica R., Hoyo J., Bassegoda A., Tzanov T., Marco M.-P., Lanceros-Méndez S., Fernández-Sánchez C. (2023). Engineering
a Point-of-Care Paper-Microfluidic Electrochemical Device Applied
to the Multiplexed Quantitative Detection of Biomarkers in Sputum. ACS Sens..

[12] Gutiérrez-Capitán M., Baldi A., Merlos A., Fernández-Sánchez C. (2022). Array of Individually
Addressable Two-Electrode Electrochemical Cells Sharing a Single Counter/Reference
Electrode for Multiplexed Enzyme Activity Measurements. Biosens. Bioelectron..

[13] Kim J., Park S., Yang H. (2025). Reference
Electrode-Free Electrochemical
Detection Using a Bare Interdigitated Array Electrode and Initial
Counterbalancing O2 Reduction. Sens. Actuators
B Chem..

[14] Horny M.-C., Lazerges M., Siaugue J.-M., Pallandre A., Rose D., Bedioui F., Deslouis C., Haghiri-Gosnet A.-M., Gamby J. (2016). Electrochemical DNA Biosensors Based
on Long-Range Electron Transfer:
Investigating the Efficiency of a Fluidic Channel Microelectrode Compared
to an Ultramicroelectrode in a Two-Electrode Setup. Lab Chip.

[15] Liu Y., Arjun A. M., Webb S., Wolfe M., Chávez J. L., Swami N. S. (2024). Redox Cycling-Based Signal Amplification at Alkanethiol
Modified Nanoporous Gold Interdigitated Microelectrodes. Anal. Chim. Acta.

[16] Mruthunjaya A. K. V., Chatelier R. C., Torriero A. A. J. (2024). Calibration-Free Electrochemical
Sensor to Monitor Factor-Xa Inhibitors at the Point-of-Care Anticoagulation
Therapy. Talanta.

[17] Borchers J. S., Campbell C. R., Van Scoy S. B., Clark M. J., Anand R. K. (2021). Redox Cycling
at an Array of Interdigitated Bipolar Electrodes for Enhanced Sensitivity
in Biosensing. ChemElectroChem.

[18] Sarkar S., Mathwig K., Kang S., Nieuwenhuis Ab. F., Lemay S. G. (2014). Redox Cycling without Reference Electrodes. Analyst.

[19] Mampallil D., Mathwig K., Kang S., Lemay S. G. (2013). Redox Couples with
Unequal Diffusion Coefficients: Effect on Redox Cycling. Anal. Chem..

[20] Pathirathna P., Balla R. J., Amemiya S. (2018). Nanogap-Based Electrochemical Measurements
at Double-Carbon-Fiber Ultramicroelectrodes. Anal. Chem..

[21] Hammond J. L., Gross A. J., Estrela P., Iniesta J., Green S. J., Winlove C. P., Winyard P. G., Benjamin N., Marken F. (2014). Cysteine-Cystine
Redox Cycling in a Gold–Gold Dual-Plate Generator-Collector
Microtrench Sensor. Anal. Chem..

[22] Zafarani H. R., Mathwig K., Sudhölter E. J.
R., Rassaei L. (2016). Electrochemical
Redox Cycling in a New Nanogap Sensor: Design and Simulation. J. Electroanal. Chem..

[23] Dotan T., Nazarenko M., Atiya Y., Shacham-Diamand Y. (2023). Transport
Effects in Biochip Sensors with Redox Cycling Amplification. Electrochim. Acta.

[24] Abe H., Yabu H., Kunikata R., Suda A., Matsudaira M., Matsue T. (2020). Redox Cycling-Based
Electrochemical CMOS Imaging Sensor
for Real Time and Selective Imaging of Redox Analytes. Sens. Actuators B Chem..

[25] White H. S., McKelvey K. (2018). Redox Cycling in Nanogap
Electrochemical Cells. Curr. Opin. Electrochem..

[26] Daruházi L., Tokuda K., Farsang G. (1989). Cyclic Voltammetry
for Reversible
Redox-Electrode Reactions in Thin-Layer Cells with Closely Separated
Working and Auxiliary Electrodes of the Same Size. J. Electroanal. Chem. Interfacial Electrochem..

[27] Yamamoto S., Uno S. (2018). Redox Cycling Realized
in Paper-Based Biochemical Sensor for Selective
Detection of Reversible Redox Molecules Without Micro/Nano Fabrication
Process. Sensors.

[28] Plana D., Jones F. G. E., Dryfe R. A. W. (2010). The Voltammetric Response of Bipolar
Cells: Reversible Electron Transfer. J. Electroanal.
Chem..

[29] Laborda E., López-Asanza J., Molina A. (2023). Theoretical Framework and Guidelines
for the Cyclic Voltammetry of Closed Bipolar Cells. Anal. Chem..

[30] López-Asanza J., Martínez-Ortiz F., Molina A., Laborda E. (2025). Square Wave
Voltammetry for Two-Polarized Electrode Electrochemical Detection:
A Theoretical-Experimental Study. ACS Electrochem..

[31] Mruthunjaya A. K. V., Hodges A. M., Chatelier R. C., Torriero A. A. J. (2023). Calibration-Free
Disposable Electrochemical Sensor with Co-Facing Electrodes: Theory
and Characterisation with Fixed and Changing Mediator Concentration. Electrochim. Acta.

[32] Dotan T., Jog A., Shacham-Diamand Y. (2024). Electrical
Modeling of an Electrochemical
Sensor Operating at the Redox Cycling Mode. Electrochim. Acta.

[33] Guajardo
Yévenes C. F., Ngamchana S., Surareungchai W. (2023). Steady-State
Currents of Planar Interdigitated Electrode Arrays in Shallow Cells. J. Electroanal. Chem..

[34] Hüske M., Stockmann R., Offenhäusser A., Wolfrum B. (2014). Redox Cycling in Nanoporous
Electrochemical Devices. Nanoscale.

[35] Han D., Zaino L. P., Fu K., Bohn P. W. (2016). Redox Cycling in
Nanopore-Confined Recessed Dual-Ring Electrode Arrays. J. Phys. Chem. C.

[36] Odijk M., Olthuis W., Dam V. A. T., van
den Berg A. (2008). Simulation
of Redox-Cycling Phenomena at Interdigitated Array (IDA) Electrodes:
Amplification and Selectivity. Electroanalysis.

[37] Molina A., Serna C., Camacho L. (1995). Conditions
of Applicability of the
Superposition Principle in Potential Multipulse Techniques: Implications
in the Study of Microelectrodes. J. Electroanal.
Chem..

[38] Molina A., Gonzalez J., Henstridge M. C., Compton R. G. (2011). Voltammetry of Electrochemically
Reversible Systems at Electrodes of Any Geometry: A General, Explicit
Analytical Characterization. J. Phys. Chem.
C.

[39] Wang L., Xie L., Song Y., Liu X., Zhang H., He X. (2023). Identifying
Cathode and Anode Polarizations during Practical High-rate Charging/Discharging
in Different Li-ion Pouch Batteries. Battery
Energy.

[40] Guidelli R., Compton R. G., Feliu J. M., Gileadi E., Lipkowski J., Schmickler W., Trasatti S. (2014). Defining the Transfer Coefficient
in Electrochemistry: An Assessment (IUPAC Technical Report). Pure Appl. Chem..

[41] Molina, A. ; González, J. Pulse Voltammetry in Physical Electrochemistry and Electroanalysis; Springer International Publishing: Cham, 2016; . 10.1007/978-3-319-21251-7.

[42] Hernández-Tovar J. V., Martínez-García A. J., López-Tenés M., Martínez-Ortiz F., Molina A., González J. (2025). From Semi-Infinite
to Thin-Layer Diffusion–Effects of Finite Mass Transport on
the Electrochemical Response of Redox Probes: Implications for Electroanalytical
Measurements. Anal. Chem..

[43] Hernández-Tovar J. V., Martínez-García A. J., López-Tenés M., Martínez-Ortiz F., Molina A., González J. (2025). Cyclic Staircase
Voltammetry and Cyclic Voltammetry in Current and Charge Modes for
the Analysis of the Responses of Fast Redox Probes under Finite Diffusion. Electrochim. Acta.

[200] Galyamin D., Laborda E., Esquivel J. P., Gonzalez J., Sabate N. (2024). Unveiling the Effect of Paper Matrix
on the Electrochemical
Response of Diffusive Redox Probes. Sensors
and Actuators Reports.

[44] Pajkossy T., Ceblin M. U., Mészáros G. (2021). Dynamic Electrochemical
Impedance Spectroscopy for the Charge Transfer Rate Measurement of
the Ferro/Ferricyanide Redox Couple on Gold. J. Electroanal. Chem..

