# Supplementary material for: Redox Cycling in Two-Polarized Electrode Sensors: Diagnostic Insights Guided by Theory
Source: ACS Meas Sci Au. 2026 Mar 20;6(2):553–63. doi: 10.1021/acsmeasuresciau.6c00024 (PMC13087961; doi:10.1021/acsmeasuresciau.6c00024)
Supplement: Supplementary file 1 [file tg6c00024_si_001.pdf]

Supporting Information

Redox Cycling in Two-Polarized Electrode Sensors: Diagnostic Insights Guided by Theory

Javier López-Asanza, Antonio Jesús Martínez-García, José Víctor Hernández-Tovar, Joaquín González, Angela Molina, Eduardo Laborda*

Departamento de Química Física, Facultad de Química, Regional Campus of International Excellence "Campus Mare Nostrum", Universidad de Murcia, 30100 Murcia, Spain

* Corresponding author:

Tel: +34 868 88 7433

Fax: +34 868 88 4148

Email: elaborda@um.es

SI1. Formulation of the boundary value problem of redox cycling in two-polarized electrode systems

The boundary value problem (bvp) corresponding to the system shown in Figure (1) of the main text, when a constant potential pulse, *E*, is applied, is given by:

$$\frac{\partial c_{\mathrm{R}}(x,t)}{\partial t} = D\frac{\partial^2 c_{\mathrm{R}}(x,t)}{\partial x^2}$$
$$\frac{\partial c_{\mathrm{O}}(x,t)}{\partial t} = D\frac{\partial^2 c_{\mathrm{O}}(x,t)}{\partial x^2} \quad \text{(S1)}$$

$t=0,\ x \geq 0\ \leq L:\ \ c_{\mathrm{R}}(x,t) = c_{\mathrm{R}}^{*},\ c_{\mathrm{O}}(x,t) = c_{\mathrm{O}}^{*}$ (S2)

$t > 0, x = 0:$

$$\left(\frac{\partial c_{\mathrm{O}}}{\partial x}\right)_{x=0} = -\left(\frac{\partial c_{\mathrm{R}}}{\partial x}\right)_{x=0} \quad \text{(S3)}$$

$$c_{\mathrm{O}}^{x=0} = \mathrm{e}^{\eta_{\mathrm{WE}}} c_{\mathrm{R}}^{x=0} \quad \text{(S4)}$$

$t > 0, x = L:$

$$\left(\frac{\partial c_{\mathrm{O}}}{\partial x}\right)_{x=L} = -\left(\frac{\partial c_{\mathrm{R}}}{\partial x}\right)_{x=L} \quad \text{(S5)}$$

$$c_{\mathrm{O}}^{x=L} = \mathrm{e}^{\eta_{\mathrm{CE}}} c_{\mathrm{R}}^{x=L} \quad \text{(S6)}$$

with $c_{\mathrm{i}}^{\mathrm{s}}$ being the interfacial concentration of species i $(\equiv \mathrm{O, R})$ and:

$$\eta_{\mathrm{WE}} = \frac{nF}{RT}(E_{\mathrm{WE}} - E_{\mathrm{O/R}}^{0'}) \ \ \text{(a)}$$
$$\eta_{\mathrm{CE}} = \frac{nF}{RT}(E_{\mathrm{CE}} - E_{\mathrm{O/R}}^{0'}) \ \ \text{(b)} \quad \text{(S7)}$$

where the unknown potential differences at the working and counter electrode-solution interfaces are related by:

$$E = E_{\mathrm{WE}} - E_{\mathrm{CE}} \quad \text{(S8)}$$

and the current across the two interfaces must be equal:

$$I = I_{WE} = I_{CE} \quad \text{(S9)}$$

By considering the equation (S9), the following relationship between the currents at the working and counter electrodes can be established:

$$I_{\mathrm{WE}}\left(=I\right) = nFAD\left(\frac{\partial c_{\mathrm{R}}}{\partial x}\right)_{x=0} = -nFAD\left(\frac{\partial c_{\mathrm{O}}}{\partial x}\right)_{x=0} \quad \text{(S10)}$$

$$I_{\mathrm{CE}}\left(=I\right) = nFAD\left(\frac{\partial c_{\mathrm{O}}}{\partial x}\right)_{x=L} = -nFAD\left(\frac{\partial c_{\mathrm{R}}}{\partial x}\right)_{x=L} \quad \text{(S11)}$$

Considering that the diffusion coefficients of both species are identical, it follows from the conditions established in equations (S3) and (S5) that, for any values of *x* and *t*, the following relationship holds:

$$c_{\mathrm{O}}(x,t) + c_{\mathrm{R}}(x,t) = c_{\mathrm{O}}^{*} + c_{\mathrm{R}}^{*} = \zeta^{*} \ \ (0 \leq x \leq L) \quad \text{(S12)}$$

Consequently, equations (S4), (S6) and (S12) can be used to derive the expressions of the interfacial concentrations of redox species as a function of the interfacial potentials

$$c_{\mathrm{O}}^{x=0}=\frac{\zeta^{*}\mathrm{e}^{\eta_{\mathrm{WE}}}}{1+\mathrm{e}^{\eta_{\mathrm{WE}}}} \qquad c_{\mathrm{O}}^{x=L}=\frac{\zeta^{*}\mathrm{e}^{\eta_{\mathrm{CE}}}}{1+\mathrm{e}^{\eta_{\mathrm{CE}}}}$$
$$c_{\mathrm{R}}^{x=0}=\frac{\zeta^{*}}{1+\mathrm{e}^{\eta_{\mathrm{WE}}}} \qquad c_{\mathrm{R}}^{x=L}=\frac{\zeta^{*}}{1+\mathrm{e}^{\eta_{\mathrm{CE}}}} \tag{S13}$$

By applying the Laplace transform to equation (S1) and solving the resulting differential equation, the following expressions for the transformed concentrations are obtained:

$$\overline{c_i}=A_i\mathrm{e}^{\sqrt{\frac{s}{D}}x}+B_i\mathrm{e}^{-\sqrt{\frac{s}{D}}x}+\frac{c_i^{*}}{s} \quad (\mathrm{i}\equiv\mathrm{R,O}) \tag{S14}$$

where A_i and B_i are constants to be determined, and s is the Laplace variable. From equations (S9), (S10) and (S11), it follows that the fluxes of a given species at $x = 0$ and $x = L$ are equal:

$$\overline{J_{x,i}}=D\left(\frac{\partial\overline{c_{\mathrm{i}}}}{\partial x}\right)_{x=0}=D\left(\frac{\partial\overline{c_{\mathrm{i}}}}{\partial x}\right)_{x=L} \tag{S15}$$

Therefore, from equations (S14) and (S15), the following expressions for A_i and B_i are obtained,

$$A_{\mathrm{i}}=\frac{\overline{J_{x,\mathrm{i}}}}{D\sqrt{\frac{s}{D}}}\frac{1-\mathrm{e}^{-\sqrt{\frac{s}{D}}L}}{2\sinh(\sqrt{\frac{s}{D}}L)}$$
$$B_{\mathrm{i}}=\frac{\overline{J_{x,\mathrm{i}}}}{D\sqrt{\frac{s}{D}}}\frac{\mathrm{e}^{\sqrt{\frac{s}{D}}L}-1}{2\sinh(\sqrt{\frac{s}{D}}L)} \tag{S16}$$

From equation (S14) and the expressions for A_i and B_i (eqs (S16)), it follows that at $x = L/2$, the Laplace-transformed concentration of species i is given by:

$$\overline{c_{\mathrm{i}}}(L/2,s)=\frac{c_{\mathrm{i}}^{*}}{s} \tag{S17}$$

Consequently, the problem can be addressed by considering that, since both species involved are consumed and regenerated at the same rate at the two electrodes, their concentrations at $x = L/2$ remain constant at their initial values for any value of t :

$$c_{\mathrm{O}}^{x=L/2}=c_{\mathrm{O}}^{*}$$
$$c_{\mathrm{R}}^{x=L/2}=c_{\mathrm{R}}^{*} \tag{S18}$$

Furthermore, from equation (S14) one can derive that:

$$c_{\mathrm{i}}^{x=0}+c_{\mathrm{i}}^{x=L}=2c_{\mathrm{i}}^{*} \tag{S19}$$

Consequently, based on the Nernstian boundary conditions at both electrodes (eqs. (S4) and (S6)), equation (S12) and equation (S19), it can be concluded that the interfacial concentrations depend solely on the applied potential as follows:

Working Electrode

$$c_{\mathrm{R}}^{x=0} = c_{\mathrm{R}}^{*}\left(1 - f(\eta)\right)$$

$$c_{\mathrm{O}}^{x=0} = c_{\mathrm{R}}^{*}\left(f(\eta) + \varepsilon\right)$$

$$E_{\mathrm{WE}} = E_{\mathrm{O/R}}^{0'} + \frac{RT}{nF}\ln\left(\frac{f(\eta)+\varepsilon}{1-f(\eta)}\right)$$

Counter Electrode

$$c_{\mathrm{R}}^{x=L} = c_{\mathrm{R}}^{*}\left(1 + f(\eta)\right)$$

$$c_{\mathrm{O}}^{x=L} = c_{R}^{*}\left(\varepsilon - f(\eta)\right) \tag{S20}$$

$$E_{\mathrm{CE}} = E_{\mathrm{O/R}}^{0'} + \frac{RT}{nF}\ln\left(\frac{\varepsilon - f(\eta)}{1+f(\eta)}\right)$$

where

$$f(\eta) = \frac{\varepsilon + 1 - \sqrt{\left(\varepsilon+1\right)^{2} - 4\varepsilon\tanh^{2}(\eta/2)}}{2\tanh(\eta/2)} \tag{S21}$$

with

$$\eta = \frac{nF}{RT}E \tag{S22}$$

$$\varepsilon = \frac{c_{\mathrm{O}}^{*}}{c_{\mathrm{R}}^{*}} \tag{S23}$$

SI2. Constant potential pulse

Based on equation (S20), it can be deduced that the interfacial potentials—and consequently, the interfacial concentrations at both electrodes—are time-independent. The problem of diffusion between two parallel planes separated by a distance *L*, each maintaining fixed concentrations at the boundaries, has been addressed extensively in various contexts [1,2]. Following the classical solution approach, the resulting expressions for the concentration profiles of both species are obtained as follows:

$$
\begin{aligned}
c_{\mathrm{R}} &= c_{\mathrm{R}}^{x=0} + (c_{\mathrm{R}}^{x=L} - c_{\mathrm{R}}^{x=0})\left(\frac{x}{L} + \frac{1}{\pi}\sum_{n=1}^{\infty} \mathrm{e}^{\frac{-(2n)^2\pi^2 Dt}{L^2}} \frac{\sin\left(\frac{2n\pi x}{L}\right)}{n}\right) \\
c_{\mathrm{O}} &= c_{\mathrm{O}}^{x=0} + (c_{\mathrm{O}}^{x=L} - c_{\mathrm{O}}^{x=0})\left(\frac{x}{L} + \frac{1}{\pi}\sum_{n=1}^{\infty} \mathrm{e}^{\frac{-(2n)^2\pi^2 Dt}{L^2}} \frac{\sin\left(\frac{2n\pi x}{L}\right)}{n}\right)
\end{aligned} \tag{S24}
$$

By differentiating the concentration profiles given in Eq. (S24) at the electrode boundaries *x*=0 and *x*=*L*, the interfacial concentration gradients at both electrodes are directly obtained:

$$
\begin{aligned}
\left(\frac{\partial c_{\mathrm{O}}}{\partial x}\right)_{x=0} &= \frac{c_{\mathrm{O}}^{*} - c_{\mathrm{O}}^{x=0}}{\delta} \qquad & \left(\frac{\partial c_{\mathrm{O}}}{\partial x}\right)_{x=L} &= \frac{c_{\mathrm{O}}^{*} - c_{\mathrm{O}}^{x=L}}{\delta} \\
\left(\frac{\partial c_{\mathrm{R}}}{\partial x}\right)_{x=0} &= \frac{c_{R}^{*} - c_{R}^{x=0}}{\delta} \qquad & \left(\frac{\partial c_{\mathrm{R}}}{\partial x}\right)_{x=L} &= \frac{c_{R}^{*} - c_{R}^{x=L}}{\delta}
\end{aligned} \tag{S25}
$$

where δ represents the diffusion layer [3]

$$
\delta = \frac{\sqrt{\pi D t}}{1 + 2\sum_{j=1}^{\infty} \mathrm{e}^{-\frac{j^2 (L/2)^2}{Dt}}} \tag{S26}
$$

Based on the methodology described in [4] and from equations (S10), (S11) and (S25), the following expressions relating the surface concentrations to the current can be derived:

$$
\begin{aligned}
&\text{Working Electrode} \qquad & &\text{Counter Electrode} \\
&c_{\mathrm{R}}^{x=0} = c_{\mathrm{R}}^{*}(1 - I_{\mathrm{N}}) \qquad & &c_{\mathrm{R}}^{x=L} = c_{\mathrm{R}}^{*}(1 + I_{\mathrm{N}}) \\
&c_{\mathrm{O}}^{x=0} = c_{\mathrm{R}}^{*}(I_{\mathrm{N}} + \varepsilon) \qquad & &c_{\mathrm{O}}^{x=L} = c_{\mathrm{R}}^{*}(\varepsilon - I_{\mathrm{N}})
\end{aligned} \tag{S27}
$$

where:

$$
I_{\mathrm{N}} = \frac{I\delta}{nFADc_{\mathrm{R}}^{*}} \tag{S28}
$$

Combining the Nernstian conditions in (S3) and (S5), with equations (S27), the following expressions are obtained for the interfacial potentials:

$$
E_{\mathrm{WE}} = E_{\mathrm{O/R}}^{0'} + \frac{RT}{nF}\ln\left(\frac{I_{\mathrm{N}} + \varepsilon}{1 - I_{\mathrm{N}}}\right) \tag{S29}
$$

$$
E_{\mathrm{CE}} = E_{\mathrm{O/R}}^{0'} + \frac{RT}{nF}\ln\left(\frac{\varepsilon - I_{\mathrm{N}}}{1 + I_{\mathrm{N}}}\right) \tag{S30}
$$

Taking into account eqn. (S8), the following relationship between the applied potential and the current is deduced:

$$E = E_{\mathrm{WE}} - E_{\mathrm{CE}} = \frac{RT}{nF}\ln\left(\frac{\left(\varepsilon + I_{\mathrm{N}}\right)\left(1+I_{\mathrm{N}}\right)}{\left(\varepsilon - I_{\mathrm{N}}\right)\left(1-I_{\mathrm{N}}\right)}\right) \quad \text{(S31)}$$

By analogy with Eqs. (S20), the following expression for the *I-E* curve is obtained:

$$I = n\,FAc_{\mathrm{R}}^{*}\sqrt{\frac{D}{\pi t}}\left(1+2\sum_{j=1}^{\infty}\mathrm{e}^{-\frac{j^{2}(L/2)^{2}}{Dt}}\right)f(\eta) \quad \text{(S32)}$$

From expression (S31), it is demonstrated that the normalized current (I_{N}) tends to −1 as the applied potential approaches negative infinity, and to 1 as it approaches positive infinity. Then the value of the half-wave potential, $E_{1/2}$, is immediately extracted by imposing the condition $I_{\mathrm{N}} = 0$:

$$E_{1/2} = 0 \quad \text{(S33)}$$

From expression (S31), $E_{1/4}$ and $E_{3/4}$, are directly obtained by setting the condition $I_{\mathrm{N}} = -0.5$ and $I_{\mathrm{N}} = 0.5$:

$$E_{1/4} = \frac{RT}{nF}\ln\left(\frac{2\varepsilon - 1}{3(2\varepsilon + 1)}\right)$$
$$E_{3/4} = \frac{RT}{nF}\ln\left(\frac{3(2\varepsilon + 1)}{2\varepsilon - 1}\right) \quad \text{(S34)}$$

And from equations (S34), the following expression for $E_{3/4} - E_{1/4}$ is derived:

$$E_{3/4} - E_{1/4} = \frac{2RT}{nF}\ln\left(\frac{3(2\varepsilon + 1)}{2\varepsilon - 1}\right) \quad \text{(S35)}$$

SI3. Second potential pulse

Now let us consider the introduction of a second potential pulse, E_2, applied at $t = \tau_1$ and maintained over the interval $\tau_1 \le t \le \tau_1 + \tau_2$, where τ_1 and τ_2 denote the durations of the first and second pulses, respectively. The bvp corresponding to the second pulse is defined as follows:

$$\begin{aligned} \frac{\partial c_{\rm R}^{(2)}(x,t)}{\partial t} &= D\frac{\partial^2 c_{\rm R}^{(2)}(x,t)}{\partial x^2} \\ \frac{\partial c_{\rm O}^{(2)}(x,t)}{\partial t} &= D\frac{\partial^2 c_{\rm O}^{(2)}(x,t)}{\partial x^2} \end{aligned} \qquad \text{(S36)}$$

$$t = \tau_1,\ \ 0 \le x \le L:\ c_{\rm R}^{(2)} = c_{\rm R}^{(1)},\ c_{\rm O}^{(2)} = c_{\rm O}^{(1)} \qquad \text{(S37)}$$

$t > \tau_1$, $x = 0$ and $x = L$:

$$\begin{aligned} &\left(\frac{\partial c_{\rm O}^{(2)}}{\partial x}\right)_{x=0} = -\left(\frac{\partial c_{\rm R}^{(2)}}{\partial x}\right)_{x=0} && \left(\frac{\partial c_{\rm O}^{(2)}}{\partial x}\right)_{x=L} = -\left(\frac{\partial c_{\rm R}^{(2)}}{\partial x}\right)_{x=L} \\ &c_{\rm O}^{x=0(2)} = {\rm e}^{\eta_{2,\rm WE}} c_{\rm R}^{x=0(2)} && c_{\rm O}^{x=L\,(2)} = {\rm e}^{\eta_{2,\rm CE}} c_{\rm R}^{x=L\,(2)} \end{aligned} \qquad \text{(S38)}$$

with

$$E_2 = E_{2,\rm WE} - E_{2,\rm CE} \qquad \text{(S39)}$$

$$I_2 = I_{2,\rm WE} = I_{2,\rm CE} \qquad \text{(S40)}$$

$c_{\rm i}^{(1)}(x,t)$ $({\rm i} \equiv {\rm O, R})$ being the solutions of the first potential pulse and:

$$\begin{aligned} \eta_{2,\rm WE} &= \frac{nF}{RT}(E_{2,\rm WE} - E_{\rm O/R}^{0'}) \\ \eta_{2,\rm CE} &= \frac{nF}{RT}(E_{2,\rm CE} - E_{\rm O/R}^{0'}) \end{aligned} \qquad \text{(S41)}$$

Taking into account that the diffusion operator in (S1) is linear, the solutions corresponding to the second potential pulse can be written as linear combinations of solutions:

$$\begin{aligned} c_{\rm R}^{(2)}(x,t) &= c_{\rm R}^{(1)}(x,t) + \tilde{c}_{\rm R}^{(2)}(x,t_2) \\ c_{\rm O}^{(2)}(x,t) &= c_{\rm O}^{(1)}(x,t) + \tilde{c}_{\rm O}^{(2)}(x,t_2) \end{aligned} \qquad \text{(S42)}$$

where $t_2 = t - \tau_1$ (so that $0 \le t_2 \le \tau_2$) and $\tilde{c}_{\rm i}^{(2)}(x,t_2)$ are the unknown partial solutions of the bvp resulting from introducing eqs. (S42) into (S36)-(S38):

$$\begin{aligned} \frac{\partial \tilde{c}_{\rm R}^{(2)}(x,t_2)}{\partial t_2} &= D\frac{\partial^2 \tilde{c}_{\rm R}^{(2)}(x,t_2)}{\partial x^2} \\ \frac{\partial \tilde{c}_{\rm O}^{(2)}(x,t_2)}{\partial t_2} &= D\frac{\partial^2 \tilde{c}_{\rm O}^{(2)}(x,t_2)}{\partial x^2} \end{aligned} \qquad \text{(S43)}$$

$$t_2 = 0,\ \ 0 \le x \le L:\ \tilde{c}_{\rm R}^{(2)} = 0,\ \tilde{c}_{\rm O}^{(2)} = 0 \qquad \text{(S44)}$$

$t_2 > 0$, $x = 0$ and $x = L$:

$$\begin{aligned} &\left(\frac{\partial \tilde{c}_{\rm O}^{(2)}}{\partial x}\right)_{x=0} = -\left(\frac{\partial \tilde{c}_{\rm R}^{(2)}}{\partial x}\right)_{x=0} && \left(\frac{\partial \tilde{c}_{\rm O}^{(2)}}{\partial x}\right)_{x=L} = -\left(\frac{\partial \tilde{c}_{\rm R}^{(2)}}{\partial x}\right)_{x=L} \\ &\tilde{c}_{\rm O}^{x=0(2)} = {\rm e}^{\eta_{2,\rm WE}} \tilde{c}_{\rm R}^{x=0(2)} + \left({\rm e}^{\eta_{2,\rm WE}} c_{\rm R}^{x=0(1)} - c_{\rm O}^{x=0(1)}\right) && \tilde{c}_{\rm O}^{x={\rm L}(2)} = {\rm e}^{\eta_{2,\rm CE}} \tilde{c}_{\rm R}^{x=L(2)} + \left({\rm e}^{\eta_{2,\rm CE}} c_{\rm R}^{x=L(1)} - c_{\rm O}^{x=L(1)}\right) \end{aligned}$$

(S45)

As the values of $c_{\mathrm{i}}^{x=0(1)}$ and $c_{\mathrm{i}}^{x=L(1)}$ $(\mathrm{i} \equiv \mathrm{O}, \mathrm{R})$ are independent of time (eqns.(S20)), the mathematical problem of $\tilde{c}_{\mathrm{i}}^{(2)}(x,t_2)$ is formally identical to that of the first pulse with null initial conditions. Thus, the solution for $\tilde{c}_{\mathrm{i}}^{(2)}(x,t_2)$ is obtained by following the same procedure described for the first pulse:

$$\tilde{c}_{\mathrm{R}}^{(2)} = \tilde{c}_{\mathrm{R}}^{x=0(2)} + \left(\tilde{c}_{\mathrm{R}}^{x=L(2)} - \tilde{c}_{\mathrm{R}}^{x=0(2)}\right)\left(\frac{x}{L} + \frac{1}{\pi}\sum_{n=1}^{\infty} \mathrm{e}^{\frac{-(2n)^2\pi^2 D t_2}{L^2}} \frac{\sin\left(\frac{2n\pi x}{L}\right)}{n}\right)$$
$$\tilde{c}_{\mathrm{O}}^{(2)} = \tilde{c}_{\mathrm{O}}^{x=0(2)} + \left(\tilde{c}_{\mathrm{O}}^{x=L(2)} - \tilde{c}_{\mathrm{O}}^{x=0(2)}\right)\left(\frac{x}{L} + \frac{1}{\pi}\sum_{n=1}^{\infty} \mathrm{e}^{\frac{-(2n)^2\pi^2 D t_2}{L^2}} \frac{\sin\left(\frac{2n\pi x}{L}\right)}{n}\right) \quad \text{(S46)}$$

with

$$\tilde{c}_{i}^{x=0(2)} = c_{\mathrm{i}}^{x=0(2)} - c_{\mathrm{i}}^{x=0(1)}$$
$$\tilde{c}_{i}^{x=L(2)} = c_{\mathrm{i}}^{x=L(2)} - c_{\mathrm{i}}^{x=L(1)} \quad \text{(S47)}$$

Hence, the solutions for the concentration gradients at the surface of the electrodes have the following form:

Working Electrode

$$\left(\frac{\partial c_{\mathrm{O}}}{\partial x}\right)_{x=0} = \frac{c_{\mathrm{O}}^{*} - c_{\mathrm{O}}^{x=0(1)}}{\delta(\tau_1 + t_2)} + \frac{c_{\mathrm{O}}^{x=0(1)} - c_{\mathrm{O}}^{x=0(2)}}{\delta(t_2)}$$
$$\left(\frac{\partial c_{\mathrm{R}}}{\partial x}\right)_{x=0} = \frac{c_{R}^{*} - c_{R}^{x=0(1)}}{\delta(\tau_1 + t_2)} + \frac{c_{R}^{x=0(1)} - c_{R}^{x=0(2)}}{\delta(t_2)}$$

Counter Electrode

$$\left(\frac{\partial c_{\mathrm{O}}}{\partial x}\right)_{x=L} = \frac{c_{\mathrm{O}}^{*} - c_{\mathrm{O}}^{x=L}}{\delta(\tau_1 + t_2)} + \frac{c_{\mathrm{O}}^{x=L(1)} - c_{\mathrm{O}}^{x=L(2)}}{\delta(t_2)}$$
$$\left(\frac{\partial c_{\mathrm{R}}}{\partial x}\right)_{x=L} = \frac{c_{R}^{*} - c_{R}^{x=L}}{\delta(\tau_1 + t_2)} + \frac{c_{R}^{x=L(1)} - c_{R}^{x=L(2)}}{\delta(t_2)} \quad \text{(S48)}$$

From these expressions (S48), together with the relationships between the current and the surface gradients of the redox species (eqns. (S10) and (S11)), the following expressions are immediately deduced for the interfacial concentrations:

Working Electrode

$$c_{\mathrm{R}}^{x=0(2)} = c_{\mathrm{R}}^{*}\left[1 - \left(I_{1,\mathrm{N}} + \frac{\delta(t_2)}{\delta(\tau_1 + t_2)}\left(I_{2,\mathrm{N}} - I_{1,\mathrm{N}}\right)\right)\right]$$
$$c_{\mathrm{O}}^{x=0(2)} = c_{\mathrm{R}}^{*}\left(\varepsilon + I_{1,\mathrm{N}} + \frac{\delta(t_2)}{\delta(\tau_1 + t_2)}\left(I_{2,\mathrm{N}} - I_{1,\mathrm{N}}\right)\right)$$

Counter Electrode

$$c_{\mathrm{R}}^{x=L(2)} = c_{\mathrm{R}}^{*}\left(1 + I_{1,\mathrm{N}} + \frac{\delta(t_2)}{\delta(\tau_1 + t_2)}\left(I_{2,\mathrm{N}} - I_{1,\mathrm{N}}\right)\right)$$
$$c_{\mathrm{O}}^{x=L(2)} = c_{\mathrm{R}}^{*}\left[\varepsilon - \left(I_{1,\mathrm{N}} + \frac{\delta(t_2)}{\delta(\tau_1 + t_2)}\left(I_{2,\mathrm{N}} - I_{1,\mathrm{N}}\right)\right)\right] \quad \text{(S49)}$$

and for the interfacial potentials:

$$E_{2,\mathrm{WE}} = E_{\mathrm{O/R}}^{0'} + \frac{RT}{nF}\ln\left(\frac{\varepsilon + I_{1,\mathrm{N}} + \frac{\delta(t_2)}{\delta(\tau_1 + t_2)}\left(I_{2,\mathrm{N}} - I_{1,\mathrm{N}}\right)}{1 - \left(I_{1,\mathrm{N}} + \frac{\delta(t_2)}{\delta(\tau_1 + t_2)}\left(I_{2,\mathrm{N}} - I_{1,\mathrm{N}}\right)\right)}\right) \quad \text{(S50)}$$

$$E_{2,\mathrm{CE}} = E_{\mathrm{O/R}}^{0'} + \frac{RT}{nF}\ln\left(\frac{\varepsilon - \left(I_{1,\mathrm{N}} + \frac{\delta(t_2)}{\delta(\tau_1 + t_2)}\left(I_{2,\mathrm{N}} - I_{1,\mathrm{N}}\right)\right)}{1 + I_{1,\mathrm{N}} + \frac{\delta(t_2)}{\delta(\tau_1 + t_2)}\left(I_{2,\mathrm{N}} - I_{1,\mathrm{N}}\right)}\right) \quad \text{(S51)}$$

with:

$$I_{\mathrm{m,N}} = \frac{I_{\mathrm{i}}\, \delta\left(\tau_1 + t_2\right)}{n\, FAD\, c_{\mathrm{R}}^{*}} \quad (\mathrm{m} = 1{,}2) \tag{S52}$$

Taking into account eqn. (S8), the following relationship between the applied potential and the current is deduced:

$$E_2 = E_{\mathrm{2,WE}} - E_{\mathrm{2,CE}} = \frac{RT}{nF} \ln \left(\frac{\left(\varepsilon + I_{\mathrm{1,N}} + \frac{\delta(t_2)}{\delta(\tau_1 + t_2)}\left(I_{\mathrm{2,N}} - I_{\mathrm{1,N}}\right)\right)\left(1 + I_{\mathrm{1,N}} + \frac{\delta(t_2)}{\delta(\tau_1 + t_2)}\left(I_{\mathrm{2,N}} - I_{\mathrm{1,N}}\right)\right)}{\left[1 - \left(I_{\mathrm{1,N}} + \frac{\delta(t_2)}{\delta(\tau_1 + t_2)}\left(I_{\mathrm{2,N}} - I_{\mathrm{1,N}}\right)\right)\right]\left[\varepsilon - \left(I_{\mathrm{1,N}} + \frac{\delta(t_2)}{\delta(\tau_1 + t_2)}\left(I_{\mathrm{2,N}} - I_{\mathrm{1,N}}\right)\right)\right]} \right) \tag{S53}$$

so that, by analogy with (S31), it is deduced that:

$$\begin{aligned} f(\eta_2) &= I_{\mathrm{1,N}} + \frac{\delta(t_2)}{\delta(\tau_1 + t_2)}\left(I_{\mathrm{2,N}} - I_{\mathrm{1,N}}\right) \\ &= \frac{\varepsilon + 1 - \sqrt{\left(\varepsilon + 1\right)^2 - 4\varepsilon \tanh^2(\eta_2 / 2)}}{2 \tanh(\eta_2 / 2)} \end{aligned} \tag{S54}$$

with:

$$\eta_2 = \frac{nF}{RT} E_2 \tag{S55}$$

in such a way that the current response in the second potential pulse is given by:

$$I_2 = n\, FA\, c_{\mathrm{R}}^{*} D \left(\frac{f(\eta_1)}{\delta(\tau_1 + t_2)} + \frac{\left(f(\eta_2) - f(\eta_1)\right)}{\delta(t_2)} \right) \tag{S56}$$

and the time-independent interfacial concentrations and interfacial potentials by:

(S57)

Working Electrode	Counter Electrode
$c_{\mathrm{R}}^{x=0(2)} = c_{\mathrm{R}}^{*}\left(1 - f(\eta_2)\right)$	$c_{\mathrm{R}}^{x=L(2)} = c_{\mathrm{R}}^{*}(1 + f(\eta_2))$
$c_{\mathrm{O}}^{x=0(2)} = c_{\mathrm{R}}^{*}(f(\eta_2) + \varepsilon)$	$c_{\mathrm{O}}^{x=L(2)} = c_{\mathrm{R}}^{*}\left(\varepsilon - f(\eta_2)\right)$
$E_{\mathrm{2,WE}} = E_{\mathrm{O/R}}^{0'} + \frac{RT}{nF}\ln\left(\frac{f(\eta_2) + \varepsilon}{1 - f(\eta_2)}\right)$	$E_{\mathrm{2,CE}} = E_{\mathrm{O/R}}^{0'} + \frac{RT}{nF}\ln\left(\frac{\varepsilon - f(\eta_2)}{1 + f(\eta_2)}\right)$

SI4. Any p-th potential pulse

When considering the application of successive potential pulses, E_1, E_2, … E_p, the linearity of the problem enables us to propose that the solutions of the p-th pulse can be written as:

$$\begin{aligned} c_\mathrm{R}^{(\mathrm{p})}(x,t) &= c_\mathrm{R}^{(\mathrm{p}-1)}(x,t) + \tilde{c}_\mathrm{R}^{(\mathrm{p})}(x,t_\mathrm{p}) \\ c_\mathrm{O}^{(\mathrm{p})}(x,t) &= c_\mathrm{O}^{(\mathrm{p}-1)}(x,t) + \tilde{c}_\mathrm{O}^{(\mathrm{p})}(x,t_\mathrm{p}) \end{aligned} \tag{S58}$$

Let $c_\mathrm{i}^{(\mathrm{p})}$ $(\mathrm{i} \equiv \mathrm{O, R})$ denote the solutions corresponding to the p-th pulse, and $c_\mathrm{i}^{(\mathrm{p}-1)}(x,t)$ those of the previous pulse (p–1), which are assumed to share the same form as the solution obtained for the second pulse. Under this assumption, it can be shown that the corresponding bvp is equivalent, and thus the solutions $\tilde{c}_\mathrm{i}^{(\mathrm{p})}$ are identical to those derived for the second pulse (see Figure S2):

$$\begin{aligned} c_\mathrm{R}^{(\mathrm{p})} &= c_\mathrm{R}^{x=0(\mathrm{p})} + (c_\mathrm{R}^{x=L(\mathrm{p})} - c_\mathrm{R}^{x=0(\mathrm{p})})\left(\frac{x}{L} + \frac{1}{\pi}\sum_{n=1}^{\infty} \mathrm{e}^{\frac{-(2n)^2\pi^2 D t_\mathrm{p}}{L^2}} \frac{\sin\left(\frac{2n\pi x}{L}\right)}{n}\right) \\ c_\mathrm{O}^{(\mathrm{p})} &= c_\mathrm{O}^{x=0(\mathrm{p})} + (c_\mathrm{O}^{x=L(\mathrm{p})} - c_\mathrm{O}^{x=0(\mathrm{p})})\left(\frac{x}{L} + \frac{1}{\pi}\sum_{n=1}^{\infty} \mathrm{e}^{\frac{-(2n)^2\pi^2 D t_\mathrm{p}}{L^2}} \frac{\sin\left(\frac{2n\pi x}{L}\right)}{n}\right) \end{aligned} \tag{S59}$$

with

$$\begin{aligned} c_i^{x=0(\mathrm{p})} &= c_\mathrm{i}^{x=0(\mathrm{p})} - c_\mathrm{i}^{x=0(\mathrm{p}-1)} \\ c_i^{x=L(\mathrm{p})} &= c_\mathrm{i}^{x=L(\mathrm{p})} - c_\mathrm{i}^{x=L(\mathrm{p}-1)} \end{aligned} \tag{S60}$$

leading to the following relationship between the applied potential and the current:

$$E_\mathrm{p} = E_\mathrm{p,WE} - E_\mathrm{p,CE} = \frac{RT}{nF}\ln\left(\frac{\left(\varepsilon + I_\mathrm{1,N} + \sum_{\mathrm{m}=2}^{\mathrm{p}} \beta_\mathrm{m}\left(I_\mathrm{m,N} - I_\mathrm{m-1,N}\right)\right)\left(1 + I_\mathrm{1,N} + \sum_{\mathrm{m}=2}^{\mathrm{p}} \beta_\mathrm{m}\left(I_\mathrm{m,N} - I_\mathrm{m-1,N}\right)\right)}{\left[1 - \left(I_\mathrm{1,N} + \sum_{\mathrm{m}=2}^{\mathrm{p}} \beta_\mathrm{m}\left(I_\mathrm{m,N} - I_\mathrm{m-1,N}\right)\right)\right]\left[\varepsilon - \left(I_\mathrm{1,N} + \sum_{\mathrm{m}=2}^{\mathrm{p}} \beta_\mathrm{m}\left(I_\mathrm{m,N} - I_\mathrm{m-1,N}\right)\right)\right]}\right) \tag{S61}$$

with:

$$I_\mathrm{m,N} = \frac{I_\mathrm{i}\,\delta(t_{m,p})}{n F A D c_R^*} \tag{S62}$$

$$\beta_\mathrm{m} = \sqrt{\frac{\delta(t_\mathrm{m,p})}{\delta(t_\mathrm{1,p})}} \tag{S63}$$

$$t_\mathrm{m,p} = \begin{cases} \sum_{\mathrm{j=m}}^{\mathrm{p}-1} \tau_\mathrm{j} + t_\mathrm{p} & \text{if } \mathrm{m} < \mathrm{p} \\ t_\mathrm{p} & \text{if } \mathrm{m} = \mathrm{p} \end{cases} \tag{S64}$$

with τ_j being the duration of the j-th pulse. Thus, the following general expression for the current response of a p-th pulse is obtained:

$$I_{\mathrm{p}} = nFAc_{\mathrm{R}}^{*}D\sum_{\mathrm{m}=1}^{\mathrm{p}}\frac{1}{\delta\;(t_{\mathrm{m,p}})}\left(f(\eta_{\mathrm{m}}) - f(\eta_{\mathrm{m}-1})\right) \qquad \text{(S65)}$$

where

$$f(\eta_0) = 0$$
$$f(\eta_{\mathrm{m}>0}) = \frac{\varepsilon + 1 - \sqrt{(\varepsilon+1)^2 - 4\varepsilon\tanh^2(\eta_m/2)}}{2\tanh(\eta_m/2)} \qquad \text{(S66)}$$

with

$$\eta_{\mathrm{m}} = \frac{nF}{RT}E_{\mathrm{m}} \qquad \text{(S67)}$$

Also, analogously to the first and second pulses, the following expressions hold for the interfacial concentrations and for the interfacial potentials:

Working Electrode

$$c_{\mathrm{R}}^{x=0(p)} = c_{\mathrm{R}}^{*}\left(1 - f(\eta_{\mathrm{p}})\right)$$
$$c_{\mathrm{O}}^{x=0(p)} = c_{\mathrm{R}}^{*}(f(\eta_{\mathrm{p}}) + \varepsilon)$$
$$E_{\mathrm{p,WE}} = E_{\mathrm{O/R}}^{0'} + \frac{RT}{nF}\ln\left(\frac{f(\eta_{\mathrm{p}}) + \varepsilon}{1 - f(\eta_{\mathrm{p}})}\right)$$

Counter Electrode

$$c_{\mathrm{R}}^{x=L(p)} = c_{\mathrm{R}}^{*}(1 + f(\eta_{\mathrm{p}}))$$
$$c_{\mathrm{O}}^{x=L(p)} = c_{\mathrm{R}}^{*}\left(\varepsilon - f(\eta_{\mathrm{p}})\right) \qquad \text{(S68)}$$
$$E_{\mathrm{p,CE}} = E_{\mathrm{O/R}}^{0'} + \frac{RT}{nF}\ln\left(\frac{\varepsilon - f(\eta_{\mathrm{p}})}{1 + f(\eta_{\mathrm{p}})}\right)$$

SI5. Interfacial potentials and concentrations

The voltammetric features and peculiarities discussed in the main text can be rationalized by examining the interfacial potentials and surface concentrations of the electroactive species. Understanding how the applied potential is distributed between the two electrodes is key to fully understand the system and provides practical insight for designing experiments and interpreting the distinctive behaviors. As can be inferred from Eqs. (S68), the interfacial potentials and concentrations depend solely on ε and on the applied potential, being independent of Λ_{CV}. Therefore, the trends described below are valid for any electrode spacing or scan rate.

Figures S1 shows the variation of E_{WE} and E_{CE} and the corresponding normalized interfacial concentrations during the forward CV scan for $\varepsilon = 1$ and $\varepsilon = 5$. As shown in Figure S1a, when the concentrations of the reduced and oxidized forms are equal ($\varepsilon = 1$), the potential is equally 'distributed' between the electrodes such that $E_{\mathrm{WE}} = -E_{\mathrm{CE}}$. At the beginning of the CV experiment (most negative potentials), the WE potential correspond to the full reduction of O at the WE (see Figure S1b), while the CE potential to the full oxidation of R. As the applied potential is scanned towards more positive values, so does the WE potential while the CE shifts to negatives values. Thus, at some point of the experiment, the current reverses so that the WE become the anode and CE the cathode. Under steady state conditions, such point correspond to the equilibrium potential: $E = E_{1/2} = 0$. Subsequently, at positive enough potentials, the depletion of the reagent takes places at each electrode; since the complete depletion of each redox species at one electrode necessarily corresponds to full regeneration at the opposing electrode, the sum of concentration of O and R at the WE ($x = 0$) and the CE ($x = \mathrm{L}$) is $2c_{\mathrm{O}}^{*}$ and $2c_{\mathrm{R}}^{*}$, respectively (eqs. (S19)).

When $\varepsilon > 1$ (e.g., $\varepsilon = 5$ in Figures S1c and S1d), the potential distribution between the two interfaces is no longer symmetric. For negative E values, the interfacial potential at the WE (blue line) remains nearly constant, while the CE potential (orange line) takes more positives values than in the case $\varepsilon = 1$. This behavior reflects the reduced ability of species R to sustain the current, rendering the anodic process at the CE the current-limiting step. This is clearly reflected in the surfaces concentrations (Fig. S1d): only species R is fully depleted at the corresponding electrode surface, while the surface concentration of O varies between $c_{\mathrm{O}}^{*} + c_{\mathrm{R}}^{*}$ and $c_{\mathrm{O}}^{*} - c_{\mathrm{R}}^{*}$. As the applied potential approaches positive values, the distribution switches: the oxidation of R at the WE becomes the limiting step, in such a way that the CE interfacial potential stabilizes while the WE potential shifts towards more positive values. It is worth noting that the

slope of the variation of the interfacial potential of the current-limiting interface is steeper for $\varepsilon > 1$ (Figure S1c) than for $\varepsilon = 1$ (Figure S1a), which means that the 'effective' scan rate is larger in the former case; consequently, the time scale required for the system to reach the steady state conditions becomes longer.

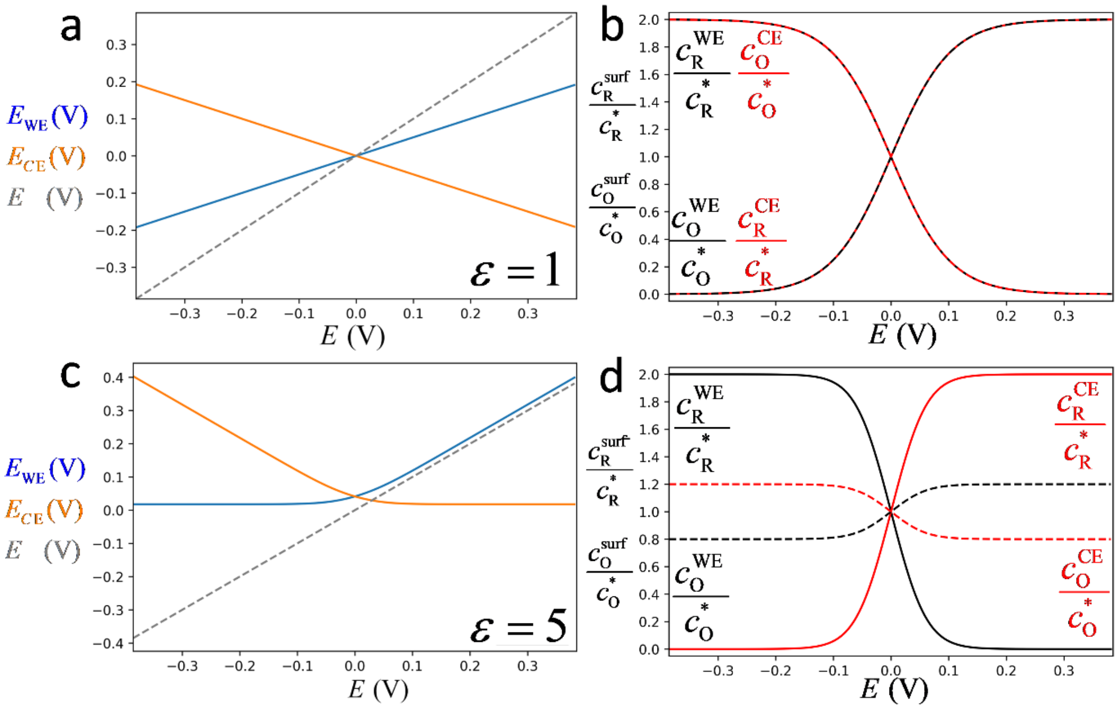

Figure S1. Variation of **(a,c)** the interfacial potentials and **(b,d)** interfacial concentrations during the forward CV scan for ε=1 and ε=5. (a,c) Dashed grey lines represent the externally applied potential.

SI6. Theoretical concentration profiles

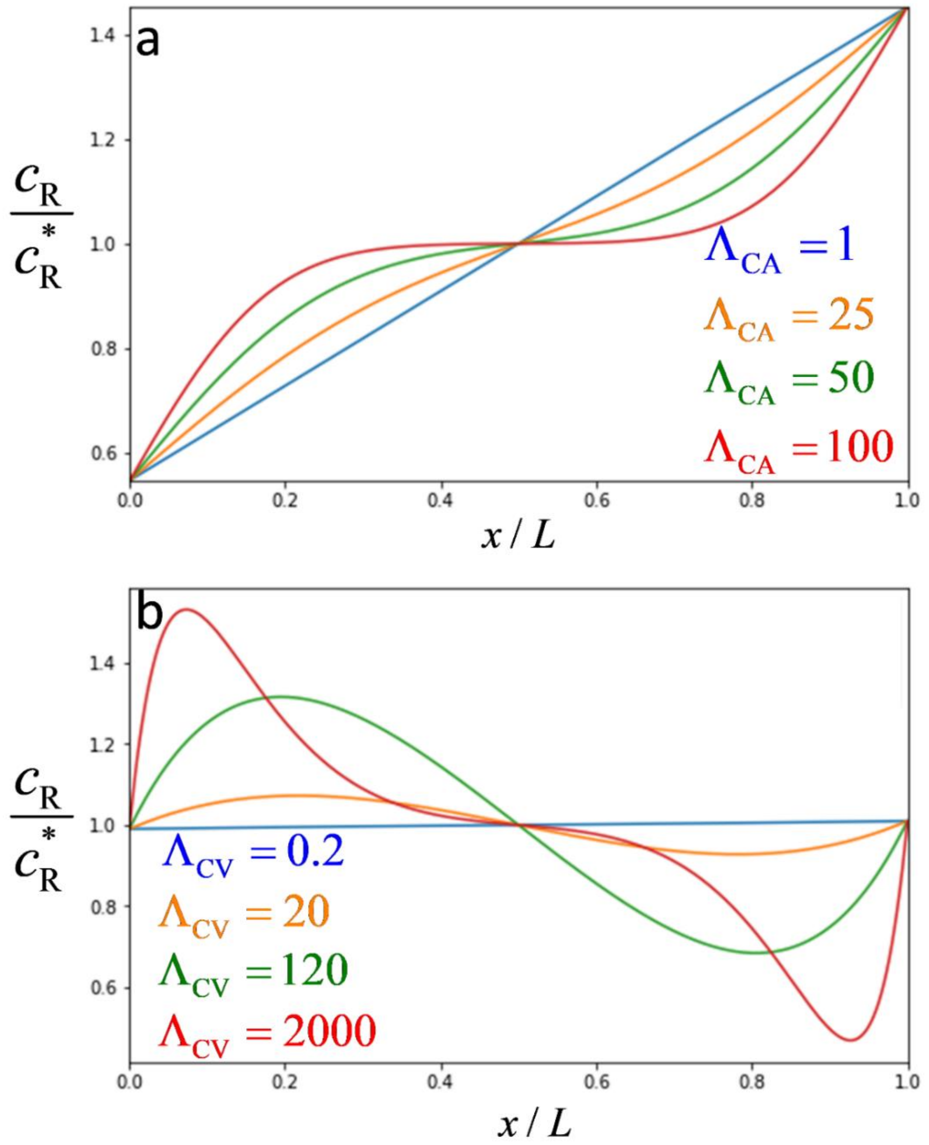

Figure S2. Normalized concentration profiles of species R (eq. (S58)) corresponding to **(a)** a chronoamperometric experiment under diffusion-limited conditions at different values of Λ_{CA} and to **(b)** the cyclic voltammetry forward scan at $E = 0$ for different Λ_{CV} -values.

References

(1) H.S. Carslaw; J.C. Jaeger. *Conduction of Heat in Solids*, 2nd ed.; Oxford University Press, 1959.

(2) J.Crank. *The Mathematics of Diffusion*, 2nd ed.; Oxford University Press, 1979.

(3) Hernández-Tovar, J. V.; Martínez-García, A. J.; López-Tenés, M.; Martínez-Ortiz, F.; Molina, A.; González, J. From Semi-Infinite to Thin-Layer Diffusion–Effects of Finite Mass Transport on the Electrochemical Response of Redox Probes: Implications for Electroanalytical Measurements. *Anal. Chem.* **2025**, *97* (5), 2941–2951. https://doi.org/10.1021/acs.analchem.4c05744.

(4) Laborda, E.; López-Asanza, J.; Molina, A. Theoretical Framework and Guidelines for the Cyclic Voltammetry of Closed Bipolar Cells. *Anal. Chem.* **2023**, *95* (47), 17311–17317. https://doi.org/10.1021/acs.analchem.3c03480.
